# Supplementary material for: Hypertension induced by pregnancy and neonatal outcome: Results from a retrospective cohort study in preterm under 34 weeks
Source: PLoS One. 2021 Aug 18;16(8):e0255783. doi: 10.1371/journal.pone.0255783 (PMC8372928; doi:10.1371/journal.pone.0255783)
Supplement: Attachment — Submitted filename: PONE-D-20-32227_reviewer.pdf [file pone.0255783.s001.pdf]

PLOS ONE

HYPERTENSION INDUCED BY PREGNANCY AND NEONATAL OUTCOME: A RETROSPECTIVE COHORT STUDY

--Manuscript Draft--

**Manuscript Number:**	PONE-D-20-32227
**Article Type:**	Research Article
**Full Title:**	HYPERTENSION INDUCED BY PREGNANCY AND NEONATAL OUTCOME: A RETROSPECTIVE COHORT STUDY
**Short Title:**	HYPERTENSION INDUCED BY PREGNANCY AND NEONATAL OUTCOME
**Corresponding Author:**	MARTA DAVID ROCHA, M.D<br>Secretaria de Estado da Saúde do Distrito Federal<br>Brasília, DISTRITO FEDERAL BRAZIL
**Keywords:**	Hypertension, Pregnancy, Newborn, Premature, Mortality, Neonates Outcomes
**Abstract:**	Objective: The present study seeks to assess the impact of gestational hypertensive syndromes on premature newborns and to establish the main morbidities and mortality in the neonatal period and at 18 months.<br>Study Design: A retrospective observational study was carried out with premature newborns of gestational age between 24 and 33 weeks and 6 days born alive in the of Hospital Materno Infantil de Brasília, in the period from January 1, 2014, to July 31, 2019.<br>Results: In total, 308 infants were born to hypertensive mothers and 387 to normotensive mothers. Newborns of hypertensive mothers had a higher risk of small for gestacional age (OR 8.1; CI 95% 5.2 - 12.6; p <0.01). Newborns of mothers with hypertension presented more necrotizing enterocolitis (OR 3.8; CI 95% 3.8-8.6). Survival was better in newborns of normotensive mothers, (OR 0.7; 95% CI 0.5 - 0.9; p <0.01). In the follow-up clinic, survival at 18 months of chronological age was similar between groups.<br>Conclusion: Arterial hypertension during pregnancy can increase the risk of low weight, small babies for gestational age, deaths in the neonatal period and enterocolitis, with no differences in weight and survival at 18 months of chronological age
**Order of Authors:**	MARTA DAVID ROCHA, M.D
	Paulo Roberto Margotto
	Karina Nascimento
	Maria Rita Novaes
**Additional Information:**	
**Question**	**Response**
**Financial Disclosure**<br><br>Enter a financial disclosure statement that describes the sources of funding for the work included in this submission. Review the submission guidelines for detailed requirements. View published research articles from *PLOS ONE* for specific examples.<br><br>This statement is required for submission and **will appear in the published article** if the submission is accepted. Please make	The authors received no specific funding for this work.

Powered by Editorial Manager® and ProduXion Manager® from Aries Systems Corporation

sure it is accurate.<br><br>**Unfunded studies**<br>Enter: *The author(s) received no specific funding for this work.*<br><br>**Funded studies**<br>Enter a statement with the following details:<br>• Initials of the authors who received each award<br>• Grant numbers awarded to each author<br>• The full name of each funder<br>• URL of each funder website<br>• Did the sponsors or funders play any role in the study design, data collection and analysis, decision to publish, or preparation of the manuscript?<br>• **NO** - Include this sentence at the end of your statement: *The funders had no role in study design, data collection and analysis, decision to publish, or preparation of the manuscript.*<br>• **YES** - Specify the role(s) played.<br><br>* typeset	
**Competing Interests**<br><br>Use the instructions below to enter a competing interest statement for this submission. On behalf of all authors, disclose any competing interests that could be perceived to bias this work—acknowledging all financial support and any other relevant financial or non-financial competing interests.<br><br>This statement **will appear in the published article** if the submission is accepted. Please make sure it is accurate. View published research articles from *PLOS ONE* for specific examples.	NO authors have competing interests<br>Enter: The authors have declared that no competing interests exist.

**NO authors have competing interests**<br><br>Enter: *The authors have declared that no competing interests exist.*<br><br>**Authors with competing interests**<br><br>Enter competing interest details beginning with this statement:<br><br>*I have read the journal's policy and the authors of this manuscript have the following competing interests: [insert competing interests here]*<br><br>* typeset	
**Ethics Statement**<br><br>Enter an ethics statement for this submission. This statement is required if the study involved:<br><br>• Human participants<br>• Human specimens or tissue<br>• Vertebrate animals or cephalopods<br>• Vertebrate embryos or tissues<br>• Field research<br><br>Write "N/A" if the submission does not require an ethics statement.<br><br>General guidance is provided below. Consult the submission guidelines for detailed instructions. **Make sure that all information entered here is included in the Methods section of the manuscript.**	it is a retrospective article , that use dates only from medical records. The ethics committee of the Foundation of Study and Research service to which the project is linked has accepted the exclusion of the consent term since the project developed in a Neonatal Public Unit. In this Unit all hospitalizations a term for clarifying the use of data for research and transfer to newborns assisted by the Federal District Health Department.

**Format for specific study types**<br><br>**Human Subject Research (involving human participants and/or tissue)**<br>• Give the name of the institutional review board or ethics committee that approved the study<br>• Include the approval number and/or a statement indicating approval of this research<br>• Indicate the form of consent obtained (written/oral) or the reason that consent was not obtained (e.g. the data were analyzed anonymously)<br><br>**Animal Research (involving vertebrate animals, embryos or tissues)**<br>• Provide the name of the Institutional Animal Care and Use Committee (IACUC) or other relevant ethics board that reviewed the study protocol, and indicate whether they approved this research or granted a formal waiver of ethical approval<br>• Include an approval number if one was obtained<br>• If the study involved *non-human primates*, add *additional details* about animal welfare and steps taken to ameliorate suffering<br>• If anesthesia, euthanasia, or any kind of animal sacrifice is part of the study, include briefly which substances and/or methods were applied<br><br>**Field Research**<br><br>Include the following details if this study involves the collection of plant, animal, or other materials from a natural setting:<br>• Field permit number<br>• Name of the institution or relevant body that granted permission	
**Data Availability**<br><br>Authors are required to make all data underlying the findings described fully available, without restriction, and from the time of publication. PLOS allows rare exceptions to address legal and ethical concerns. See the PLOS Data Policy and FAQ for detailed information.	No - some restrictions will apply

A Data Availability Statement describing where the data can be found is required at submission. Your answers to this question constitute the Data Availability Statement and **will be published in the article**, if accepted.<br><br>**Important:** Stating 'data available on request from the author' is not sufficient. If your data are only available upon request, select 'No' for the first question and explain your exceptional situation in the text box.<br><br>Do the authors confirm that all data underlying the findings described in their manuscript are fully available without restriction?	
**Describe where the data may be found in full sentences. If you are copying our sample text, replace any instances of XXX with the appropriate details.**<br><br>• If the data are **held or will be held in a public repository**, include URLs, accession numbers or DOIs. If this information will only be available after acceptance, indicate this by ticking the box below. For example: *All XXX files are available from the XXX database (accession number(s) XXX, XXX.).*<br>• If the data are all contained **within the manuscript and/or Supporting Information files**, enter the following: *All relevant data are within the manuscript and its Supporting Information files.*<br>• If neither of these applies but you are able to provide **details of access elsewhere**, with or without limitations, please do so. For example:<br><br>*Data cannot be shared publicly because of [XXX]. Data are available from the XXX Institutional Data Access / Ethics Committee (contact via XXX) for researchers who meet the criteria for access to confidential data.*<br><br>*The data underlying the results presented in the study are available from (include the name of the third party*	Data cannot be shared publicly because of the computer system of the Health Department of the Federal District. Data are available on the FEPECS Ethics Committee

and contact information or URL).<br>• This text is appropriate if the data are owned by a third party and authors do not have permission to share the data.<br><br>* typeset	
Additional data availability information:	Tick here if the URLs/accession numbers/DOIs will be available only after acceptance of the manuscript for publication so that we can ensure their inclusion before publication.

Manuscript
Click here to access/download;Manuscript;ARTIGO MARTA ROCHA.docx

HYPERTENSION INDUCED BY PREGNANCY AND NEONATAL OUTCOME: A RETROSPECTIVE COHORT STUDY

Marta D. Rocha de Moura [a]
Paulo R. Margotto [b]
Karina N. Costa [c]
Maria R. C. G. Novaes [d]

[a] Escola Superior de Ciências da Saúde, SMHN Conjunto A Bloco 01 Edifício FEPECS - Asa Norte, Brasília - DF, 70710-907.

[b] UTI Neonatal, Hospital Materno Infantil de Brasília, SGAS 608 A, Asa Sul , Brasília – DF , 70603900.

[c] Departamento de Medicina da Criança e do Adolescente da Universidade de Brasília, UnB - Brasília, DF, 70910-900.

[a] Escola Superior de Ciências da Saúde, SMHN Conjunto A Bloco 01 Edifício FEPECS - Asa Norte, Brasília - DF, 70710-907.

Author: Marta David Rocha de Moura, Escola Superior de Ciências da Saúde, SMHN Conjunto A Bloco 01 Edifício FEPECS - Asa Norte, Brasília - DF, 70710-907; fax +5561 20172132, email: marta.rocha@escs.edu.br.

Abstract

Objective: The present study seeks to assess the impact of gestational hypertensive syndromes on premature newborns and to establish the main morbidities and mortality in the neonatal period and at 18 months.
Study Design: A retrospective observational study was carried out with premature newborns of gestational age between 24 and 33 weeks and 6 days born alive in the of Hospital Materno Infantil de Brasília, in the period from January 1, 2014, to July 31, 2019.
Results: In total, 308 infants were born to hypertensive mothers and 387 to normotensive mothers. Newborns of hypertensive mothers had a higher risk of small for gestacional age (OR 8.1; CI 95% 5.2 - 12.6; p <0.01). Newborns of mothers with hypertension presented more necrotizing enterocolitis (OR 3.8; CI 95% 3.8-8.6). Survival was better in newborns of normotensive mothers, (OR 0.7; 95% CI 0.5 - 0.9; p <0.01). In the follow-up clinic, survival at 18 months of chronological age was similar between groups.
Conclusion: Arterial hypertension during pregnancy can increase the risk of low weight, small babies for gestational age, deaths in the neonatal period and enterocolitis, with no differences in weight and survival at 18 months of chronological age.

Keywords: Hypertension, Pregnancy, Newborn, Premature, Mortality

Introduction

Hypertensive pregnancy syndrome (GHS) is a serious complication that affects approximately 2.5 to 3.0 percent of women, increasing the risk of maternal and neonatal complications [1,2]. Worldwide, hypertensive disorders remain the leading cause of maternal mortality related to pregnancy. In the United States, hypertensive disorders contribute 7% to 12% of maternal deaths related to pregnancy [1-3].

Hypertensive disorders of pregnancy are classified into 4 categories, as recommended by the National High Blood Pressure Education Program of the American College of Obstetrics and Gynecology (ACOG) working group: (1) chronic hypertension, (2) pre-eclampsia, (3) pre-eclampsia superimposed on chronic hypertension and (4) gestational hypertension [3,4]. Hypertension in pregnancy caused by fear of the professional's white coat is included as an additional category by some societies, such as the International Society for the Study of Hypertension in Pregnancy [3].

Hypertensive syndromes of pregnancy appear as a hypertensive condition that develops at any time after 20 weeks of pregnancy, accompanied or not by proteinuria. [3,4].

Hypertension during pregnancy cannot be prevented; therefore, the identification of maternal risk factors becomes an important obstetric mission [1]. The risk factors are associated with the development of gestational hypertension, previous history of pre-eclampsia, primiparity, obesity, family history of pre-eclampsia, number of previous pregnancies (if any) and chronic medical conditions, such as hypertension

and diabetes, maternal age greater than or equal to 40 years, obesity, diabetes mellitus and chronic kidney disease. [1-6].

The decision to terminate pregnancy must balance maternal and neonatal risks; thus, the basic objective of obstetrics is to carefully prolong pregnancy to improve the perinatal outcome without compromising maternal safety [7,8].

Some authors suggest that expectant control of pregnancy is associated with an increase in maternal complications in a pregnancy of 32 weeks or more [7-9].

The neonatal complications described range from prematurity to fetal growth restriction, the latter being the most frequent neonatal complication in newborns (NB) of hypertensive mothers [10-13].

Other morbidities described in newborns of mothers with hypertensive conditions are bronchopulmonary dysplasia (BPD), retinopathy of prematurity (ROP), sepsis and longer time on mechanical ventilation. Hematological changes such as thrombocytopenia and leukopenia are also frequently described in the literature [10,11].

Exposure to SHG may be associated with an increased risk of autism spectrum disorder (ASD) and attention deficit/hyperactivity disorder [12,14].

The aim of this study is to assess the impact of gestational hypertensive syndromes on premature newborns below 34 weeks and to identify the mortality rate and morbidities at discharge and at 18 months of corrected gestational age.

Material and methods

A retrospective cohort study was conducted in which all preterm infants, with gestational age (GA) between 24 and 33 weeks and 6 days, born alive in a maternity center in a reference center in Brasília, Distrito Federal, Brazil, at Hospital Materno, Infantil de Brasília (HMIB), and admitted to the Intensive Care Unit - Neonatal ICU of this Hospital, from January 1, 2014, to July 31, 2019, were included.

The study design followed the STROBE checklist recommendations of the Equator Network [15]. The sample size was calculated using the Epi Info program, version 7.2.2.6, considering a 10% prevalence of hypertension during pregnancy and considering a 99.9% confidence. The estimated sample size was 432 newborns. During the study period, it was possible to select 695 NBs for the study, divided into two groups, G1 - hypertensive pregnant women (G1), with 308 newborns (NB), and G2 - normotensive pregnant women (NB), with 387 NB.

Exclusion criteria were: premature infants transferred directly from the delivery room to another service, as well as deaths, twins, newborns from other services and patients with severe malformations diagnosed by skull ultrasound. All patients included in the study were followed up during hospitalization and evaluated for the outcomes of interest at 28 days of life or at discharge/death/transfer and at 18 months of corrected gestational age.

The independent variables analyzed included the following gestational data, obtained from obstetric notes and confirmed in the postpartum period: prenatal care (> 2 consultations for premature births); maternal age; maternal infection (clinical chorioamnionitis or peripartum urinary tract infection); antenatal steroid use (> one dose administered before delivery, regardless of the type of steroid); use of magnesium sulfate, diagnosis of hypertensive syndrome of pregnancy (including variations pre-eclampsia, eclampsia, HELLP syndrome and pre-eclampsia overlapping with chronic arterial hypertension); and the presence of zero (DZ) or reverse (DR) diastoles in gestational ultrasound up to 72 hours before and during delivery.

The data related to birth were: gestational age defined by the best obstetric estimate or was estimated by examining the newborn, performed immediately after birth or within 12 hours, using the method of New Ballard [16]. Other data analyzed were: gender; birth weight; weight and gestational age classification to define small for gestational age (SGA), according to the criteria of Lubchenco et al. [16] and Margotto [17], from 29 weeks; Apgar of the 1st and 5th minutes and need for resuscitation in the delivery room.

Regarding neonatal evolution, the independent variables analyzed were: respiratory distress syndrome (RDS) with clinical and radiological diagnosis; ventilatory assistance time (continuous positive airway pressure - nasal CPAP and/or mechanical ventilation); neonatal sepsis; brain alterations diagnosed at skull ultrasound, diagnosis of bronchopulmonary dysplasia (BPD) defined by the need for supplemental oxygen at 36 weeks of postmenstrual age, and death and/or survival at 28 days of life. Diagnosis of necrotizing enterocolitis was defined by the presence of at least one clinical sign: bilious gastric aspirate or vomiting; abdominal distension; or occult or evident blood in the stool without anal fissure. Presence of at least one radiological sign included intestinal pneumatosis, gas in the hepatobiliary system and/or pneumoperitoneum.

Skull ultrasound was performed on all newborns who survived up to 7 days. In newborns who had more than one examination, the worst ultrasound finding was considered. Cystic leukomalacia was defined

by the presence of small cavities close to the lateral ventricles, and peri-intraventricular hemorrhage was classified according to the criteria of Papile et al. [18] and considered severe when it reached grade III or IV. The examinations were performed with GE® equipment by two neonatologists who specialized in diagnostic imaging, responsible for the routine assessment of preterm infants younger than 34 weeks of gestation.

The results were analyzed using SPSS version 22.0. The mean, standard deviation and Student's t test were used to compare quantitative variables with symmetrical distribution, such as birth weight, gestational age, maternal age, Apgar score and length of hospital stay. Categorical variables were analyzed using absolute and relative frequencies and were compared using the Chi-square test with Yates correction. The calculation of odds ratios (OR) and 95% confidence intervals (CI) were used to test the association of categorical variables. This study was approved by the Research Ethics Committee by the Foundation and Teaching and Research in Health Sciences - FEPECS, of the Health Department of the Federal District under registration CAAE 58280716.9.0000.5553, approval number 2,137,741.

Results

In the period from January 1, 2014, to July 31, 2019, 1765 newborns admitted to the Neonatal ICU of the HMIB ~~during this period.~~ Of these, 695 met the inclusion criteria for the study. A total of 550 newborns were excluded for the following reasons: multiple malformations (85); gestational diabetes (106); transferred (108) and pregnant women who were not submitted to Doppler velocimetry of umbilical arteries (154). Of the 695 newborns included in the study, 308 were born to hypertensive mothers (GHA), and 387 were born to normotensive mothers (GN).

Among NBs born to ~~hypertensive mothers,~~ 69.8% (215 pregnant women) underwent ultrasound with umbilical artery Doppler fluxometry, and of these babies, 14.4% (31 pregnant women) had a normal examination. Zero diastole (DZ) occurred in 55.8% (134 pregnant women) and reverse diastole (DR) in 20.8% (50 pregnant women).

In ~~the group born of hypertensive pregnant women,~~ gestational hypertensive disease was present in 50%, pre-eclampsia in 12.5%, eclampsia in 11.3% and chronic arterial hypertension alone or superimposed on pre-eclampsia in 12.1% of pregnant women.

There was no difference between groups regarding gestational age and the use of antenatal corticosteroids. The use of magnesium sulfate was frequent in the group of hypertensive pregnant women, just as the preferred mode of delivery in this group was cesarean delivery.

Table 1 shows the morbidity and mortality of the newborns studied. The anthropometric measurements birth weight and head circumference were significantly lower in G1 DZ/DR. Table 2 shows that the G1 group had a higher risk of SGA NB (OR 8.1; CI 95% 5.2 - 12.6; p <0.01), as well as the greater the risk of being born with a weight less than 850 g (OR 3.4; 95% CI 2.2 - 5.4; p <0.01). Newborns of mothers with hypertension presented more necrotizing enterocolitis (OR 3.8; CI 95% 3.8-8.6), but resuscitation in the delivery room and the need to use surfactant did not differ between groups, nor did length of stay on mechanical ventilation, oxygen use at 28 days of life, or oxygen dependence at 36 weeks of gestational age (Table 3).

The changes in transfontanellar ultrasound ~~until~~ the first week of life, as well as the worst ultrasound image described before discharge or death, did not differ between groups. (Table 3).

Survival was better in newborns of normotensive mothers, ~~which was a protective factor against death~~ (OR 0.7; 95% CI 0.5 - 0.9; p <0.01). In the follow-up clinic, survival at 18 months of chronological age was similar between groups, with rates of 92.5% and 93.7% among hypertensive and normotensive mothers, respectively. Exclusive breastfeeding at discharge was 47.6% in the group of hypertensive women and 52.4% in the group of normotensive mothers. There were no significant differences between groups (Table 5).

Using logistic regression, the only isolated indicators for death in the studied sample were the diagnoses of reverse diastole (OR of 8.2; 95% CI 3.5 - 19.6) and GA at birth. Every~~one~~ week ~~more than~~ GA reduces the risk of death by 30% (OR 0.7; 95% CI 0.6 - 0.8).

Discussion

This study ~~shows how severe~~ neonatal outcomes ~~can be~~ in the presence of hypertension during pregnancy, especially if it is accompanied by changes in umbilical artery Doppler flowmetry. The presence of reverse diastole in the umbilical artery increases the chance of death by 8.2 (95% CI 3.5 - 19.6) in the neonatal period.

In a classic study, Villar et al. [19] found that women with pre-eclampsia and SGA had a higher risk of their newborns staying in the neonatal ICU and experiencing neonatal death after adjusting the study for the location and socioeconomic status, but this difference disappeared after adjusting for birth weight

and gestational age. This suggests that the excess risk associated with pre-eclampsia and SGA can be explained by these variables [19].

Webster et al. [20], in a systematic review with meta-analysis, suggest that antihypertensive treatment reduces the risk of severe hypertension in pregnant women with chronic hypertension, and consequently, less maternal and neonatal complications. However, the lack of randomized controlled trials does not clarify which is the best therapeutic option [20].

It was observed that most women attended prenatal care; however, normotensive mothers had fewer consultations (4.3 ± 2.2 (~~G2)~~ and 4.7 ± 2.1 (G1), p= 0.04). Adequate monitoring of the pregnant woman is extremely important in preventing both maternal and fetal morbidity and mortality.

Hypertensive pregnant women were older, with a mean age of 28.8 ± 66 compared to normotensive women, 25.7 ± 6.9 (p= 0.00). The prevalence of gestational hypertensive disease increases proportionally with the increase in maternal age and occurs more commonly in women over 34 years old; the data collected by this study demonstrate the same trend [21,22].

The use of antenatal corticosteroids above 70% in both groups reflects obstetric care; the benefits of this therapy are described in the literature in aspects such as decreased risk of neonatal death, the occurrence of respiratory distress syndrome and intraventricular hemorrhage [23].

Magnesium sulfate has been well evaluated as an effective intervention in the treatment of women with preeclampsia. A meta-analysis of data from six studies that evaluated 11,444 women with pre-eclampsia demonstrated that magnesium sulfate versus placebo or no use of anticonvulsants was able to reduce the risk of eclampsia by more than half, without affecting the incidence of fetal or neonatal death [23].

It was observed that in the studied group, 56.3% of hypertensive pregnant women used magnesium sulfate against only 5.9% of normotensive pregnant women. The low use of magnesium sulfate among normotensive women in this study is worrying. In a review published in 2017 ~~27~~ by Cochrane, it was concluded that prenatal magnesium sulfate given to women at imminent risk of premature birth substantially reduced the risk of cerebral palsy in children. The risk of cerebral palsy is almost one-third lower in children who received prenatal magnesium sulfate (OR 0.68; 95% CI 0.54 to 0.87), making evident the need to sensitize the obstetric team of the need to use magnesium sulfate in all pregnant women at risk of premature birth [24,25].

The preferred mode of delivery among hypertensive pregnant women was cesarean delivery in 65.8% of cases (OR 2.1; 95% CI 1.7 - 2.5), reflecting the need to ~~terminate pregnancy~~ due to maternal and/or fetal risk. Due to the severity of the condition, cesarean section has been the preferred mode of delivery in cases of hypertensive syndrome. However, the American College of Obstetrics and Gynecology (ACOG) recommends vaginal delivery for this group of pregnant women [2~~6~~]. It is suggested that vaginal delivery is safer than operative delivery in women with pre-eclampsia and should be attempted. The isolated diagnosis of pre-eclampsia should not be seen as an immediate indication for cesarean delivery; however, hypertensive pregnancy syndrome may be accompanied by other problems such as fetal distress and anomalous presentations, which constitute an indication for cesarean section [26].

Chorioaminionitis and the use of antibiotics were more frequent in the group of normotensive than hypertensive mothers, 26.7% and 8.8%, respectively (p <0.00). We can consider that sudden infectious conditions led to premature birth in newborns (~~NB)~~ in the control group of this study. Although the causes of approximately half of cases of prematurity are of unknown etiology, infectious conditions are classically described as risk factors for prematurity [27].

In the group born of hypertensive pregnant women, gestational hypertensive disease was present in 50% of cases, pre-eclampsia in 12.5%, eclampsia in 11.3% and chronic arterial hypertension in isolation or superimposed on pre-eclampsia in 12.1% of pregnant women. Studies have pointed to the high risk of adverse outcomes in pregnant women who have overlapping pathologies [3-5]. As it is a small sample, it was not possible to consider the subtypes diagnosed in the different groups for a more detailed analysis, as it was chosen not to stratify the samples for an individualized analysis of the cases.

However, it was observed that the newborns born to hypertensive mothers had lower birth weight (1,007.1 ± 323.7 grams) compared to controls (1346.1 ± 409.6 grams p = 0.00). The same occurred with head circumference (28.0 4.2 cm in ~~G1~~ and 29.1 cm ± 3.4 in ~~GN,~~ p <0.00). In addition, infants born to hypertensive mothers had a higher frequency of intrauterine growth restriction, being classified as small for gestational age (SGA), 55.4%, compared to the control group, 13.3% (OR 8.1; 95% CI 5.2 - 12.6). These findings are in accordance with studies that found the same association between maternal arterial hypertension and NB with growth restriction [5,12,28].

The weight difference at the time of discharge was also evident, with G1 weighing 1.4 ± 1.28 and ~~GN~~ 1.7 ± 1.4 kg; however, there was no significant difference between the groups (p= 0.68), demonstrating that NB of hypertensive mothers may take longer to reach the ideal weight for discharge and at 18 months.

When evaluating the babies again, this time at 18 months of age, an attempt was made to identify whether gestational hypertensive syndrome would be a risk factor for growth disorders in low-weight preterm infants. For this, logistic regression models were constructed, controlled by gestational age and sex, including maternal hypertension. In the classification between birth weight for gestational age, it was observed that maternal hypertension was not a risk factor for inadequate weight growth (OR = 0.54; 95% CI: 0.21 - 2.1) at 18 months of corrected age. Similar data were observed by Kiy et al. [28], in a cohort of premature infants at 18 months.

It was identified in the present study that gestational arterial hypertension increased the chance of small NB for gestational age (SGA) by approximately two and a half times (OR = 2.61; 95% CI: 1.5; 6.4).

Considering this was a retrospective study, there was a difficulty in finding adequate records of height. The ordering of the main results of this study alerts to the higher frequency of growth disorder in low birth weight preterm children born to hypertensive mothers [8,10,11].

There were no differences regarding the need for resuscitation and the diagnosis of respiratory distress syndrome, as well as the need for exogenous surfactant. Although a significantly increased risk for RDS has not been found, authors suggest that this type of association is possible [10,11].

Maternal hypertension and prematurity can lead to immaturity in the fetal gastrointestinal tract, poor vascular supply and alteration of the intestinal microbiota, which in turn can be associated with a cascade of events, culminating in the development of necrotizing enterocolitis (NEC) [29,30]. In the present study, a relevant association was observed between maternal hypertensive syndrome and neonatal NEC.

With changes in placental flow, the risk of NEC increases, a relationship already addressed in the scientific work by Vieira et al. [31], where the presence of maternal hypertension increased the risk of NEC by 2.8 times in NBs with birth weight less than 1500 g, and the maternal diagnosis of pre-eclampsia was significantly more frequent in the NB group with NEC [26,32]. In the present study, the NEC presented an OR of 3.8 (95% CI 1.7 - 8.6) [31].

There were no differences between the groups regarding the time of use of mechanical ventilation, nasal CPAP and oxygen at 28 days or 36 weeks of gestational age, demonstrating that respiratory changes are not linked to maternal pressure changes, but to complications and limitations of prematurity and low birth weight.

The literature is conflicting about the effects of pre-eclampsia and the development of retinopathy of prematurity (ROP), one of the main causes of childhood blindness worldwide. No differences in the diagnosis of ROP between groups were observed in this study [33]. Retinopathy of prematurity is initially manifested with delayed physiological vascular development of the retina, followed by aberrant vasoproliferation, and is highly correlated with extreme prematurity and low postnatal growth.

In the sample studied, there were no differences between groups regarding the presence of cerebral hemorrhage and leukomalacia. These changes can be identified by cerebral ultrasound and are associated with an increased risk of neurological development disorders, which may inform the need for greater pediatric surveillance of babies exposed to maternal hypertensive disease. Ensuring early intervention can help improve the outcome of neurological development; therefore, the neurological prognosis of these premature mothers with arterial hypertension is still very controversial [10,12].

Higher neonatal mortality was observed in the group of hypertensive mothers, confirming the results of previous studies [8,10,11]. When the results were adjusted for birth weight, the presence of hypertension was not shown to be an isolated risk factor for death (OR 1.3 of 95% CI 0.8 - 1.9). This result follows what has been described in the literature; however, if the mother presents changes in the flow in the umbilical artery with DR, the risk of death was significant (OR 10.6; 95% CI 4.6 - 24, 4).

Shen et al. [34], studying the accuracy of Doppler ultrasound parameters in the second and third trimester of hypertensive pregnancies in determining perinatal outcomes in 1,054 pregnancies, it was observed that the analysis of the bilateral uterine artery, umbilical artery and vein and velocity data of venous flow that were monitored by Doppler indicated risk of fetal death, premature termination of pregnancy and birth weight below 2,500 g, with sensitivities of 94.4% and 93.1%, in addition to specificities of 95.2% and 90.1%, respectively.

The results of this retrospective study, should be interpreted keeping in mind such limitations.which only allows the analysis of the data documented in the medical records. it was still possible to detect significant and clinically relevant differences between preterm infants of hypertensive mothers compared to normotensive mothers.

Our study determined the significant burdens of perinatal morbidity and mortality associated with maternal hypertensive syndrome in the population of newborns treated at the HMIB. We recommend careful monitoring of hypertensive pregnant women with a multidisciplinary and careful approach, guiding the follow-up of these women and their children to improve the clinical results of these newborns.

Acknowledgements

We gratefully appreciate the support of medical doctors of Neonatal Intenvise Care of Brasília Maternal Hospital.

Conflict of interest
The authors declare that they have no conflict of interests.

Author contributions
All authors were involved in the concept and design of the study. PRM and MRCGN conducted independent literature searches and quality assessment, MDRM data extraction and wrote the first draft of the manuscript. KN and PRM supervised the article selection, quality assessment and data presentation. All authors have read and approved the final manuscript and agree to be accountable for all aspects of the work. No form of payment was given to anyone to produce the manuscript.

Funding information
Not applicable.

References
1. Shah S, Gupta A. Hypertensive disorders of pregnancy. *Cardiol Clin* 2019; **37**: 345–354.
2. Umesawa M, Kobashi G. Epidemiology of hypertensive disorders in pregnancy: prevalence, risk factors, predictors and prognosis. *Hypertens Res* 2017; **40**: 213–220.
3. Brown MA, Magee LA, Kenny LC, Karumanchi SA, McCarthy FP, Saito S, *et al.* The hypertensive disorders of pregnancy: ISSHP classification, diagnosis & management recommendations for international practice. *Pregnancy Hypertens* 2018; **13**: 291–310.
4. Yu H, He Y, Mao Z, Dong W, Fu X, Lei X. Hypertensive disorders during pregnancy and elevated blood pressure in the offspring: a systematical review and meta-analysis protocol. *Medicine* 2019; **98**: e15677.
5. Nzelu D, Dumitrascu-Biris D, Kay P, Nicolaides KH, Kametas NA. Severe hypertension, preeclampsia and small for gestational age in women with chronic hypertension diagnosed before and during pregnancy. *Pregnancy Hypertens* 2018; **14**: 200–204.
6. Rodrigues Â, Barata C, Marques I, Almeida MC. Diagnosis of white coat hypertension and pregnancy outcomes. *Pregnancy Hypertens* 2018; **14**: 121–124.
7. Wang Y, Hao M, Sampson S, Xia J. Elective delivery versus expectant management for pre-eclampsia: a meta-analysis of RCTs. *Arch Gynecol Obstet* 2017; **295**: 607–622.
8. Omani-Samani R, Ranjbaran M, Amini P, Esmailzadeh A, Sepidarkish M, Almasi-Hashiani A. Adverse maternal and neonatal outcomes in women with preeclampsia in Iran. *J Matern Fetal Neonatal Med* 2019; **32**: 212–216.
9. Nisa MU, Anjum S. Elective induction versus expectant management of mild pre-eclampsia at term. *J Coll Physicians Surg Pak* 2018; **28**: 677–680.
10. Marins LR, Anizelli LB, Romanowski MD, Sarquis AL. How does preeclampsia affect neonates? Highlights in the disease’s immunity. *J Matern Fetal Neonatal Med* 2019; **32**: 1205–1212.
11. Lu C-Q, Lin J, Yuan L, Zhou J-G, Liang K, Zhong Q-H, *et al.* Pregnancy induced hypertension and outcomes in early and moderate preterm infants. *Pregnancy Hypertens* 2018; **14**: 68–71.
12. Maher GM, O'Keeffe GW, Kearney PM, Kenny LC, Dinan TG, Mattsson M, *et al.* Association of hypertensive disorders of pregnancy with risk of neurodevelopmental disorders in offspring: a systematic review and meta-analysis. *JAMA Psychiatry* 2018; **75**: 809–819.
13. Bharadwaj SK, Vishnu Bhat B, Vickneswaran V, Adhisivam B, Bobby Z, Habeebullah S. Oxidative stress, antioxidant status and neurodevelopmental outcome in neonates born to pre-eclamptic mothers. *Indian J Pediatr* 2017; **85**: 351–357.
14. Nathan HL, Seed PT, Hezelgrave NL, De Greeff A, Lawley E, Conti-Ramsden F, *et al.* Maternal and perinatal adverse outcomes in women with pre-eclampsia cared for at facility-level in South Africa: a prospective cohort study. *J Glob Health* 2018; **8**: 020401.
15. von Elm E, Altman DG, Egger M, Pocock SJ, Gotzsche PC, Vandenbroucke JP, *et al.* The strengthening the reporting of observational studies in epidemiology (STROBE) statement: guidelines for reporting observational studies. *J Clin Epidemiol* 2008; **61**: 344–349.
16. Lubchenco L, Hansman C, Dressler M, Boyd E. Intrauterine growth as estimated from liveborn birth-weight data at 24 to 42 weeks of gestation. *Pediatrics* 1963; **32**: 793–800.
17. Margotto PR. Curvas de crescimento intra-uterino: estudo de 4413 recém-nascidos únicos de gestações normais. *J Pediatr* 1995; **71**: 11–21.

18. Papile LA, Burstein J, Burstein R, Koffler H. Incidence and evolution of subependymal and intraventricular hemorrhage: A study of infants with birth weights less than 1,500 gm. *J Pediatr* 1978; **92**: 529–534.
19. Villar J, Carroli G, Wojdyla D, Abalos E, Giordano D, Ba'aqeel H, *et al*. Preeclampsia, gestational hypertension and intrauterine growth restriction, related or independent conditions? *Am J Obstet Gynecol* 2006; **194**: 921–931.
20. Webster LM, Conti-Ramsden F, Seed PT, Webb AJ, Nelson-Piercy C, Chappell LC. Impact of antihypertensive treatment on maternal and perinatal outcomes in pregnancy complicated by chronic hypertension: a systematic review and meta-analysis. *J Am Heart Assoc* 2017; **6**: e005526.
21. Shen M, Smith GN, Rodger M, White RR, Walker MC, Wen SW. Comparison of risk factors and outcomes of gestational hypertension and pre-eclampsia. *PLoS One* 2017; **12**: e0175914.
22. Deshmukh M, Patole S. Antenatal corticosteroids for neonates born before 25 weeks-a systematic review and meta-analysis. *PLoS One* 2017; **12**: e0176090.
23. Duley L, Gülmezoglu AM, Henderson-Smart DJ, Duley L. Magnesium sulphate and other anticonvulsants for women with pre-eclampsia. *Cochrane Database Syst Rev* 2010; **2010**: CD000025.
24. Jameson RA, Bernstein HB. Magnesium sulfate and novel therapies to promote neuroprotection. *Clin Perinatol* 2019; **46**: 187–201.
25. American College of Obstetricians Gynecologists, Task Force on Hypertension in Pregnancy. Hypertension in pregnancy. Report of the American college of obstetricians and gynecologists' task force on hypertension in pregnancy. *Obstet Gynecol* 2013; **122**: 1122–1131.
26. Vogel JP, Chawanpaiboon S, Moller A-B, Watananirun K, Bonet M, Lumbiganon P. The global epidemiology of preterm birth. *Best Pract Res Clin Obstet Gynaecol* 2018; **52**: 3–12.
27. Razak A, Florendo-Chin A, Banfield L, Abdul Wahab MG, McDonald S, Shah PS, *et al*. Pregnancy-induced hypertension and neonatal outcomes: a systematic review and meta-analysis. *J Perinatol* 2018; **38**: 46–53.
28. Kiy AM, Rugolo LMSS, Luca AKCD, Corrente JE. Growth of preterm low birth weight infants until 24 months corrected age: effect of maternal hypertension. *J Pediatr* 2015; **91**: 256–262.
29. Yang C-C, Tang P-L, Liu P-Y, Huang W-C, Chen Y-Y, Wang H-P, *et al*. Maternal pregnancy-induced hypertension increases subsequent neonatal necrotizing enterocolitis risk: a nationwide population-based retrospective cohort study in Taiwan. *Medicine* 2018; **97**: e11739.
30. Neu J. Necrotizing enterocolitis. *World Rev Nutr Diet* 2014; **110**: 253–263.
31. Vieira AA, David BBL, Lino RRG, Duarte LDB, Bueno AC. Avaliação dos fatores perinatais que interferem na incidência de enterocolite necrosante em recém-nascidos de muito baixo peso. *Rev Bras Ginecol Obstet* 2013; **35**: 363–367.
32. Zhu T, Zhang L, Zhao F, Qu Y, Mu D. Association of maternal hypertensive disorders with retinopathy of prematurity: a systematic review and meta-analysis. *PLoS One* 2017; **12**: e0175374.
33. Bashinsky AL. Retinopathy of prematurity. *N C Med J* 2017; **78**: 124–128.
34. Shen G, Huang Y, Jiang L, Gu J, Wang Y, Hu B. Ultrasound prediction of abnormal infant development in hypertensive pregnant women in the second and third trimester. *Sci Rep* 2017; **7**: 40429.

Table 1. Characteristics of the studied pregnant women and neonates divided considering the presence or not of hypertensive syndrome

	G1<br>n: 308 NB	G2<br>n: 387 NB	p
Maternal Age	28.8 ± 6.6 years	25.7 ± 6.9 years	0.00
Number of prenatal consultations	4.7 ± 2.1	4. 3 ± 2.2	0.04
Gestational Age	29.4± 2.5	29.6 ± 2.4	0.30
Cesarean delivery%	65.8	29.3	0.00
Urinary infection %	9.6	23.7	0.00
~~Corrioamnionitis%~~	8.8	26.7	0.00
Prenatal corticosteroids%	78.3	73.3	0.25
Magnesium Sulfate%	56.3	5.9	0.00
Birth weight (grams)	1007.0 ± 323.7	1346.2 ± 409.6	0.00
Head circumference (cm)	28.0 ± 4.2	29.1 ± 3.4	0.02
V. Mechanical time (days)	7.4 ± 16.7	5.2 ± 13.4	0.91
Nasal CPAP time (days)	4.4 ± 6.8	3.9 ± 6.3	0.20
Supplemental O2 time (days)	17.0 ± 26.3	14.1 ± 23.1	0.74
Apgar 1 min	6.1 ± 2.1	6.2 ± 2.1	0.11
Apgar 5 min	7.9 ± 1.5	7.8 ± 1.6	0.52
Hospitalization time in days	37.8 ± 30.5	38.8 ± 38.7	0.74
Weight at hospital discharge	1.4 ± 1.28	1.7 ± 1.4	0.68

G1: NB born to mothers with hypertension G2: NB born to normotensive mothers.
Student's T test; ** X2; Fisher/G1 exact test: NB born to hypertensive mothers; G2: NB born to normotensive mothers, CPAP - Continuous positive air pressure; PIG - Small for gestational age.

Table 2. Characteristics of the studied pregnant women and neonates divided considering the presence or not of hypertensive syndrome

	G1<br>n: 308 NB	G2<br>n: 387 NB	OR (IC 95%)
NB <850 g %	31.7	11.9	3.4 (2.2 to 5.4)
SGI rating%	55.4	13.3	8.1 (5.2 to 12.6)
Male %	51.2	50	1.0 (0.7 to 1.5)
Resuscitation at sunrise%	66.3	66.3	1.0 (0.7 to 1.5)
Use of surfactant%	47.1	46.7	1.0 (0.7 to 1.4)
Early sepsis%	26.7	31.5	0.8 (0.5 to 1.2)
O2 at 28 days of life%	28.7	27.8	1.0 (0.7 to 1.5)
Use of O2 at 36 without%	17.1	13.3	1.3 (0.8 to 2.2)
Necrotizing Enterocolitis%	10.4	3	3.8 (1.7 to 8.6)
Retinopathy of prematurity%	5.0	3.7	1.4 (0.5 to 3.2)
HIV 1 and 2 (%)	28.3	19	1.4 (0.6 to 3.2)
HIV 3 and 4 (%)	15.6	13.8	0.5 (0.1 to 3.0)
Leukomalacia (%)	1.4	2.9	0.5 (0.4 to 1.5)
Hyperechogenicity (%)	35.8	45.3	0.8 (0.5 to 1.3)
Normal (%)	18.9	19	0.9 (0.5 to 1.7)

HIV: Intraventricular Hemorrhage Test X2, Fisher's exact test.

Table 3. Survival at discharge and at 18 months

	G1<br>n: 308 RN	G2<br>n: 387 RN	p	OR	IC 95%
Survival to high%	64.3	73	0.04	0.7	0.5 - 0.9
Survival at 18 months (%)	92.5	93.7	0.12	1.9	0.9 – 3.9
Breastfeeding at discharge	47.6%	52.4%	0.10	0.6	0.4 – 1.1
Weight at 18 months (kg)	7.8 ± 2.7	8.2 ± 3.2	0.08		

G1: NB born to mothers with hypertension G2: NB born to normotensive mothers

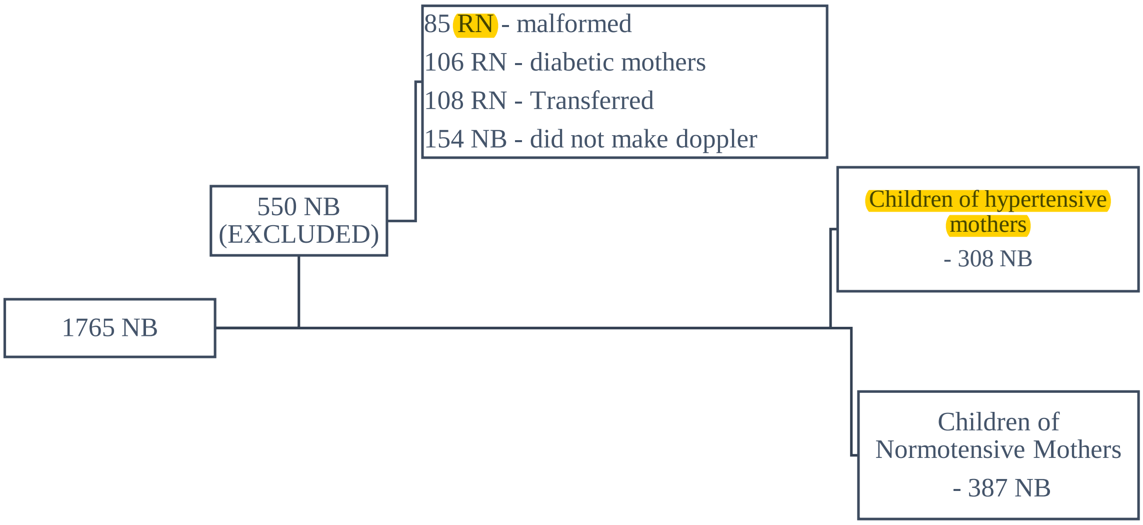

Figure 1. Design of the article
