# Supplementary material for: Hypertension induced by pregnancy and neonatal outcome: Results from a retrospective cohort study in preterm under 34 weeks
Source: PLoS One. 2021 Aug 18;16(8):e0255783. doi: 10.1371/journal.pone.0255783 (PMC8372928; doi:10.1371/journal.pone.0255783)
Supplement: Attachment — Submitted filename: PONE-D-20-32227_R1_reviewer.pdf [file pone.0255783.s002.pdf]

PLOS ONE

Hypertension induced by pregnancy and neonatal outcome: Results from a retrospective cohort study in preterm under 34 weeks.

--Manuscript Draft--

**Manuscript Number:**	PONE-D-20-32227R1
**Article Type:**	Research Article
**Full Title:**	Hypertension induced by pregnancy and neonatal outcome: Results from a retrospective cohort study in preterm under 34 weeks.
**Short Title:**	HYPERTENSION INDUCED BY PREGNANCY AND NEONATAL OUTCOME
**Corresponding Author:**	MARTA DAVID ROCHA, M.D<br>Secretaria de Estado da Saúde do Distrito Federal<br>Brasília, DISTRITO FEDERAL BRAZIL
**Keywords:**	Hypertension, Pregnancy, Newborn, Premature, Mortality, Neonates Outcomes
**Abstract:**	Abstract<br><br>Objective: The present study seeks to assess the impact of gestational hypertensive disorders on premature newborns below 34 weeks and to establish the main morbidities and mortality in the neonatal period and at 18 months.<br>Materials and methods: A retrospective observational study was carried out with 695 premature newborns of gestational age (GA) between 24 and 33 weeks and 6 days, born alive in the Neonatal ICU of Brasília's Mother and Child Hospital (HMIB), in the period from January 1, 2014, to July 31, 2019. In total, 308 infants were born to hypertensive mothers (G1) and 387 to normotensive mothers (G2). Twin pregnancies and diabetic patients with severe malformations were excluded. Outcomes during hospitalization and outcomes of interest were evaluated: respiratory distress syndrome (RDS), brain ultrasonography, diagnosis of bronchopulmonary dysplasia (BPD), diagnosis of necrotizing enterocolitis, retinopathy of prematurity, breastfeeding rate at discharge, survival at discharge and at 18 months of chronological age and relationship between weight and gestational age.<br>Results: Newborns with hypertensive mothers had significantly lower measurements of birth weight and head circumference. The G1 group had a higher risk of small babies for gestational age Small for gestational age (OR 2.4; CI 95% 1.6 – 3.6; p <0.00), as well as a greater risk of being born with a weight less than 850 g (OR 2.4; 95% CI 1.2 – 3.5; p <0.00). Newborns of mothers with hypertension presented more necrotizing enterocolitis (OR 2.0; CI 95% 1.1-3.7); however, resuscitation in the delivery room and the need to use surfactant did not differ between groups, nor did the length of stay on mechanical ventilation, or dependence on oxygen at 36 weeks of gestational age. Survival was better in newborns of normotensive mothers, and this was a protective factor against death (OR 0.7; 95% CI 0.5 - 0.9; p <0.01). In the follow-up clinic, survival at 18 months of chronological age was similar between groups, with rates of 95.3% and 92.1% among hypertensive and normotensive mothers, respectively. Exclusive breastfeeding at discharge was 73.4% in the group of hypertensive women and 77.3% in the group of normotensive mothers. There were no significant differences between groups.<br>Conclusion: Among the analyzed outcomes, arterial hypertension during pregnancy can increase the risk of low weight, small babies for gestational age (SGA), deaths in the neonatal period and enterocolitis, with no differences in weight and survival at 18 months of chronological age. Arterial hypertension presents a high risk of prematurity in the neonatal period, with no difference at 18 months of age.
**Order of Authors:**	MARTA DAVID ROCHA, M.D
	Paulo Roberto Margotto
	Karina Nascimento
	Maria Rita Novaes
**Response to Reviewers:**	Dear Reviewers,

Powered by Editorial Manager® and ProduXion Manager® from Aries Systems Corporation

Through this, we saw the submission of the manuscript, corrected and revised entitled: “Hypertension induced by pregnancy and neonatal outcome: Results from a retrospective cohort study in preterm under 34 weeks.” under the authorship of , Marta David Rocha de Moura, Paulo Roberto Margotto, Karina Nascimento and Maria Rita CG Novaes for your appreciation.
rewritten article suggestions and very pertinent comments, thus contributing to improve the quality of our article. After a detailed analysis of the comments and questions, as well as the errors pointed out and suggestions contained in the opinions sent to us, the article has undergone some changes, which are indicated below.

1. Is the manuscript technically sound, and do the data support the conclusions?

The summary and the introduction have been changed in order to accommodate the request of the reviewers. The justification, which appears in the last paragraph of the introduction, prior to the objective, was reformulated as follows to meet the reviewers' request.

2.Has the statistical analysis been performed appropriately and rigorously?

A revision was made in the Material and Methods in order to add such information.
In the first paragraph of the Material and Methods, the following excerpts were included, in order to meet the reviewer's request.

3.Have the authors made all data underlying the findings in their manuscript fully available?
I understand the importance of the requested authors. made the study database available at Dryad is an open source, community driven project that takes a unique approach to data publication and digital preservation. Being found in the register ROCHA, MARTA; Carvalho Garbi Novaes, Maria Rita; Margotto, Paulo Roberto; Nascimento Costa, Karina (2021), HYPERTENSION INDUCED BY PREGNANCY AND NEONATAL OUTCOME: A RETROSPECTIVE COHORT STUDY, Dryad, Dataset, https://doi.org/10.5061/dryad.47d7wm3cn

4.Is the manuscript presented in an intelligible fashion and written in standard English?

In addition to the corrections, the text underwent a profound grammatical revision and correction, made by an American professor of English and a biologist appointed by the University of Brasilia, in addition to having previously been sent to the American Journal Experts (AJE) where word adjustments were made. and spacing.

5.Review Comments to the Author
Reviewer #1.
Thank you for all the recommendations presented. Adjustments were made to the body of the text to meet requests. An orthographic review and adjustment in the text and abbreviations presented in the article were made. It was really unclear in the text initially sent and some terms were translated and confused in the English language. We clarify that umbilical artery dopllerfluxometry was only performed in G1 - newborns of hypertensive mothers. Exclusion criteria were better described in an attempt to clarify blind spots.

Reviewer #2:
Thank you very much for the excellent placements, the spelling and grammar was adjusted by an American proofreader appointed by the University of Brasilia. The abbreviations were standardized, there were really serious structural errors that left the term confused and poorly written. Almost the entire text was redone in order to clarify the points raised in the revised manuscript, all changes are punctuated;

Reviewer #3
Thank you for all the important recommendations, the introduction has been redone as well as the discussion has been rearranged to give more attention to the points raised. The grammar and spelling check was also done and to make the text clearer and more objective.

Reviewer #4

Powered by Editorial Manager® and ProduXion Manager® from Aries Systems Corporation

	Thanks for all the important recommendations, the title has been modified, the data errors presented have been adjusted, as well as the introduction has been shortened, the methodology has been adjusted in order to meet all the recommendations raised. The contributions were very useful and valuable.
**Additional Information:**	
**Question**	**Response**
**Financial Disclosure**<br><br>Enter a financial disclosure statement that describes the sources of funding for the work included in this submission. Review the submission guidelines for detailed requirements. View published research articles from *PLOS ONE* for specific examples.<br><br>This statement is required for submission and **will appear in the published article** if the submission is accepted. Please make sure it is accurate.<br><br>**Unfunded studies**<br>Enter: *The author(s) received no specific funding for this work.*<br><br>**Funded studies**<br>Enter a statement with the following details:<br>• Initials of the authors who received each award<br>• Grant numbers awarded to each author<br>• The full name of each funder<br>• URL of each funder website<br>• Did the sponsors or funders play any role in the study design, data collection and analysis, decision to publish, or preparation of the manuscript?<br>• **NO** - Include this sentence at the end of your statement: *The funders had no role in study design, data collection and analysis, decision to publish, or preparation of the manuscript.*<br>• **YES** - Specify the role(s) played.<br><br>* typeset	The authors received no specific funding for this work.
**Competing Interests**<br><br>Use the instructions below to enter a competing interest statement for this submission. On behalf of all authors, disclose any competing interests that could be perceived to bias this work—acknowledging all financial support	NO authors have competing interests<br>Enter: The authors have declared that no competing interests exist.

and any other relevant financial or non-financial competing interests.<br><br>This statement **will appear in the published article** if the submission is accepted. Please make sure it is accurate. View published research articles from *PLOS ONE* for specific examples.<br><br>**NO authors have competing interests**<br><br>Enter: *The authors have declared that no competing interests exist.*<br><br>**Authors with competing interests**<br><br>Enter competing interest details beginning with this statement:<br><br>*I have read the journal's policy and the authors of this manuscript have the following competing interests: [insert competing interests here]*<br><br>* typeset	
**Ethics Statement**<br><br>Enter an ethics statement for this submission. This statement is required if the study involved:<br><br>• Human participants<br>• Human specimens or tissue<br>• Vertebrate animals or cephalopods<br>• Vertebrate embryos or tissues<br>• Field research<br><br>Write "N/A" if the submission does not require an ethics statement.<br><br>General guidance is provided below. Consult the submission guidelines for detailed instructions. **Make sure that all information entered here is included in the Methods section of the manuscript.**	it is a retrospective article , that use dates only from medical records. The ethics committee of the Foundation of Study and Research service to which the project is linked has accepted the exclusion of the consent term since the project developed in a Neonatal Public Unit. In this Unit all hospitalizations a term for clarifying the use of data for research and transfer to newborns assisted by the Federal District Health Department.

Powered by Editorial Manager® and ProduXion Manager® from Aries Systems Corporation

**Format for specific study types**<br><br>**Human Subject Research (involving human participants and/or tissue)**<br>• Give the name of the institutional review board or ethics committee that approved the study<br>• Include the approval number and/or a statement indicating approval of this research<br>• Indicate the form of consent obtained (written/oral) or the reason that consent was not obtained (e.g. the data were analyzed anonymously)<br><br>**Animal Research (involving vertebrate animals, embryos or tissues)**<br>• Provide the name of the Institutional Animal Care and Use Committee (IACUC) or other relevant ethics board that reviewed the study protocol, and indicate whether they approved this research or granted a formal waiver of ethical approval<br>• Include an approval number if one was obtained<br>• If the study involved *non-human primates*, add *additional details* about animal welfare and steps taken to ameliorate suffering<br>• If anesthesia, euthanasia, or any kind of animal sacrifice is part of the study, include briefly which substances and/or methods were applied<br><br>**Field Research**<br><br>Include the following details if this study involves the collection of plant, animal, or other materials from a natural setting:<br>• Field permit number<br>• Name of the institution or relevant body that granted permission	
**Data Availability**<br><br>Authors are required to make all data underlying the findings described fully available, without restriction, and from the time of publication. PLOS allows rare exceptions to address legal and ethical concerns. See the PLOS Data Policy and FAQ for detailed information.	Yes - all data are fully available without restriction

A Data Availability Statement describing where the data can be found is required at submission. Your answers to this question constitute the Data Availability Statement and **will be published in the article**, if accepted.<br><br>**Important:** Stating 'data available on request from the author' is not sufficient. If your data are only available upon request, select 'No' for the first question and explain your exceptional situation in the text box.<br><br>Do the authors confirm that all data underlying the findings described in their manuscript are fully available without restriction?	
**Describe where the data may be found in full sentences. If you are copying our sample text, replace any instances of XXX with the appropriate details.**<br><br>• If the data are **held or will be held in a public repository**, include URLs, accession numbers or DOIs. If this information will only be available after acceptance, indicate this by ticking the box below. For example: *All XXX files are available from the XXX database (accession number(s) XXX, XXX.*).<br>• If the data are all contained **within the manuscript and/or Supporting Information files**, enter the following: *All relevant data are within the manuscript and its Supporting Information files.*<br>• If neither of these applies but you are able to provide **details of access elsewhere**, with or without limitations, please do so. For example:<br><br>*Data cannot be shared publicly because of [XXX]. Data are available from the XXX Institutional Data Access / Ethics Committee (contact via XXX) for researchers who meet the criteria for access to confidential data.*<br><br>*The data underlying the results presented in the study are available from (include the name of the third party*	I understand the importance of the requested authors. made the study database available at Dryad is an open source, community driven project that takes a unique approach to data publication and digital preservation. Being found in the register ROCHA, MARTA; Carvalho Garbi Novaes, Maria Rita; Margotto, Paulo Roberto; Nascimento Costa, Karina (2021), HYPERTENSION INDUCED BY PREGNANCY AND NEONATAL OUTCOME: A RETROSPECTIVE COHORT STUDY, Dryad, Dataset, https://doi.org/10.5061/dryad.47d7wm3cn

and contact information or URL).<br>• This text is appropriate if the data are owned by a third party and authors do not have permission to share the data.<br><br>* typeset	
Additional data availability information:	Tick here if the URLs/accession numbers/DOIs will be available only after acceptance of the manuscript for publication so that we can ensure their inclusion before publication.

Powered by Editorial Manager® and ProduXion Manager® from Aries Systems Corporation

Manuscript

Click here to access/download;Manuscript;Manuscript revewier.docx

Hypertension induced by pregnancy and neonatal outcome: Results from a retrospective cohort study in preterm under 34 weeks.

Moura, MDR [1*] , Margotto P.R.[2], Costa K.N.[3], Novaes M.R.C.G.[4]

Marta David Rocha de Moura [1]

Paulo Roberto Margotto [2]

Karina Nascimento Costa [3]

Maria Rita Carvalho Garbi Novaes [4]

[1] Assistant doctor, neonatal intensive care unit, Brasília Mother and Child Hospital , SGAS 608 A, Asa Sul , Brasília – DF , 70603900.

[2] Neonatal Senior doctor, intensive care unit, Brasília Mother and Child Hospital , SGAS 608 A, Asa Sul , Brasília – DF , 70603900.

[3] Department of Child and Adolescent Medicine, University of Brasília, **UnB** - Brasília, DF, 70910-900.

[4] Higher School of Health Sciences, SMHN Conjunto A B 01 FEPECS - Asa Norte, Brasília - DF, 70710-907.

* Corresponding author: Marta David Rocha de Moura, email: marta.rocha@escs.edu.br.

[¶] These authors contributed equally to this work.

[&] these authors also contributed equally to this work.

Abstract

Objective: The present study seeks to assess the impact of gestational hypertensive disorders on premature newborns below 34 weeks and to establish the main morbidities and mortality in the neonatal period and at 18 months.

Materials and methods: A retrospective observational study was carried out with 695 premature newborns of gestational age (GA) between 24 and 33 weeks and 6 days, born alive in the Neonatal ICU of Brasília's Mother and Child Hospital (HMIB), in the period from January 1, 2014, to July 31, 2019. In total, 308 infants were born to hypertensive

mothers (G1) and 387 to normotensive mothers (G2). Twin pregnancies and diabetic patients with severe malformations were excluded. Outcomes during hospitalization and outcomes of interest were evaluated: respiratory distress syndrome (RDS), brain ultrasonography, diagnosis of bronchopulmonary dysplasia (BPD), diagnosis of necrotizing enterocolitis, retinopathy of prematurity, breastfeeding rate at discharge, survival at discharge and at 18 months of chronological age and relationship between weight and gestational age.

Results: Newborns with hypertensive mothers had significantly lower measurements of birth weight and head circumference. The G1 group had a higher risk of small babies for gestational age Small for gestational age (OR 2.4; CI 95% 1.6 – 3.6; $p < 0.00$), as well as a greater risk of being born with a weight less than 850 g (OR 2.4; 95% CI 1.2 – 3.5; $p < 0.00$). Newborns of mothers with hypertension presented more necrotizing enterocolitis (OR 2.0; CI 95% 1.1-3.7); however, resuscitation in the delivery room and the need to use surfactant did not differ between groups, nor did the length of stay on mechanical ventilation, or dependence on oxygen at 36 weeks of gestational age. Survival was better in newborns of normotensive mothers, and this was a protective factor against death (OR 0.7; 95% CI 0.5 - 0.9; $p < 0.01$). In the follow-up clinic, survival at 18 months of chronological age was similar between groups, with rates of 95.3% and 92.1% among hypertensive and normotensive mothers, respectively. Exclusive breastfeeding at discharge was 73.4% in the group of hypertensive women and 77.3% in the group of normotensive mothers. There were no significant differences between groups.

Conclusion: Among the analyzed outcomes, arterial hypertension during pregnancy can increase the risk of low weight, small babies for gestational age (SGA), deaths in the neonatal period and enterocolitis, with no differences in weight and survival at 18 months of chronological age. Arterial hypertension presents a high risk of prematurity in the neonatal period, with no difference at 18 months of age.

Introduction

Hypertensive Disorders of Pregnancy (HDP) present a serious complication that affects approximately 2.5 to 3.0 percent of women, increasing the risk of maternal and neonatal complications [1,2]. Worldwide, hypertensive disorders remain the leading cause of maternal mortality related to pregnancy. [1]

Hypertensive disorders of pregnancy appear as a hypertensive condition that develops at any time after 20 weeks of pregnancy, accompanied or not by proteinuria. Among the manifestations of these syndromes is eclampsia, which presents with a convulsive component, and HELPP syndrome, which manifests with the presence of hemolysis, elevated liver enzymes and thrombocytopenia; HELLP is a severe form of preeclampsia and not a separate disorder [3,4].

Hypertensive Disorders of Pregnancy cannot be prevented; therefore, the identification of maternal risk factors becomes an important obstetric mission [1]. The risk factors ~~are~~ associated with the development of gestational hypertension, previous history of pre-eclampsia, primiparity, obesity, family history of pre-eclampsia, number of previous pregnancies (if any) and chronic medical conditions, such as hypertension and diabetes, maternal age greater than or equal to 40 years, obesity, diabetes mellitus, chronic kidney disease, systemic lupus erythematosus, presence of antiphospholipid, multiple pregnancies and high altitude. In addition, it is important to highlight the action of ~~kidney~~ transplant of live kidneys, which can increase the risk of developing pre-eclampsia in the recipients by up to six-fold more than in non-transplanted women [1-6].

The decision to terminate pregnancy must balance maternal and neonatal risks; thus, the basic objective of obstetrics is to carefully prolong pregnancy to improve the perinatal outcome without compromising maternal safety [7,8]. Some authors suggest that expectant control of pregnancy is associated with an increase in maternal complications in a pregnancy of 32 weeks or more [7-9].

The neonatal complications described here range from prematurity to fetal growth restriction. The latter is the most frequent neonatal complication in newborns (NB) with hypertensive mothers [10,12,13,14]. Perinatal mortality rates in growth-restricted neonates are 6 to 10 times that of those with normal growth [2,5]. Doppler ultrasonography in fetuses of hypertensive mothers is a way of assessing the severity of intrauterine growth restriction and intrauterine monitoring allows the disease progression to be observed non-invasively. [11].

Other morbidities described in newborns of mothers with hypertensive conditions are bronchopulmonary dysplasia (BPD), retinopathy of prematurity (ROP), sepsis and longer time on mechanical ventilation. Hematological changes such as thrombocytopenia and leukopenia are also frequently described in the literature [10].

Exposure to HDP may be associated with an increased risk of autism spectrum disorder (ASD) and attention deficit/hyperactivity disorder [12,13]. These findings

highlight the need for greater pediatric surveillance of newborns exposed to HDP to allow early interventions that can improve neurological development outcomes.

The aim of this study is to assess the impact of gestational hypertensive disorders on premature newborns below 34 weeks and to identify the mortality rate and morbidities at discharge and at 18 months of corrected gestational age.

Material and methods

Study design, setting and population.

This retrospective cohort study included preterm infants, with gestational age (GA) between 24 and 33 weeks and 6 days, born alive in a maternity center in Brasília, Federal District, Brazil, at the Mother and Child Hospital (Hospital Materno Infantil de Brasília, HMIB), and admitted to the Neonatal Intensive Care Unit of this Hospital, from January 1, 2014, to July 31, 2019.

HMIB is a Teaching and Referral Hospital in Federal District. The hospital This hospital was opened in1964 and it is a referral center since 1988 for neonates, women, and surgical children as tertiary hospital. Having a medical residency program in the areas of pediatrics, neonatology, pediatric intensive care, obstetrics, fetal medicine and pediatric surgery

A total of 18768 deliveries in the institution during the specified period. Preterm born in the period was 1765 newborns. (Figure 1)

Exclusion criteria were premature infants transferred directly from the delivery room to another service, as well as deaths found in the delivery room, twins, newborns from other services and patients with severe malformations, diabetics mothers and NB that have not undergone umbilical artery doppler flowmetry. All patients included in the study were followed up during hospitalization and evaluated for the outcomes of interest at 28 days of life or at discharge/death/transfer and at 18 months of corrected gestational age.

Sample size calculations and statistical analysis

In The study design followed the STROBE checklist recommendations of the Equator Network [15]. Sample size calculations and statistical analysis. Sample size calculations were done in Epi Info TM version 7.2.2.6 (Centers for Disease Control and Prevention (CDC); USA) at 80% power and 95% confidence interval. Based on a 10%

prevalence of hypertension during pregnancy, considering a 99.9% confidence level, 90% power and a 1:1 ratio (exposed and unexposed). The estimated sample size was 432 newborns, including 216 newborns with hypertensive mothers and 216 newborns with normotensive mothers. During the study period, it was possible to select 695 NBs for the study, divided into two groups, G1 - hypertensive pregnant women, with 308 newborns, and G2 - normotensive pregnant women with 387 newborns.

Operational definitions

Hypertension in pregnancy was defined as systolic blood pressure (BP) ≥140 mmHg and/or a diastolic BP ≥ 90 mmHg. Preeclampsia was defined as characterized by a BP of 140/90 mm Hg or greater after 20 weeks' gestation in a woman with previously normal BP and who have proteinuria. Eclampsia was defined as seizures that cannot be attributable to other causes, in a woman with preeclampsia. In this study, hypertensive disorders of pregnancy were classified as preeclampsia, gestational hypertension, chronic hypertension and preeclampsia superimposed on chronic hypertension. [3]

Data variables for the study order to assess the fetal outcomes of hypertensive disorders of pregnancy, data was obtained from the medical records of the study subjects. analyzed included the following gestational data, obtained from obstetric notes and confirmed in the postpartum period: prenatal care (> 2 consultations for premature births); maternal age; maternal infection (clinical chorioamnionitis or peripartum urinary tract infection); antenatal steroid use (> one dose administered before delivery, regardless of the type of steroid); use of magnesium sulfate, diagnosis of hypertensive disease of pregnancy (including variations of pre-eclampsia, eclampsia, HELLP syndrome and pre-eclampsia overlapping with chronic arterial hypertension); and the presence of Absent end-diastolic velocity waveform in gestational ultrasound up to 72 hours before and during delivery.

The data related to birth were gestational age defined by the best obstetric estimate, preferably the precise date of the last menstruation, followed by early obstetric ultrasound (up to the 18th week of pregnancy). In the absence of such data, gestational age (GA) was estimated by examining the newborn, performed immediately after birth or within 12 hours, using the method of New Ballard [15]. Other data analyzed were gender; birth weight; weight and gestational age classification to define small for gestational age (SGA), according to the criteria of Lubchenco et al. [16] and Margotto [17], from 29 weeks; Apgar of the 1st and 5th minutes; need for resuscitation in the delivery room

defined using positive pressure ventilation under a mask; and the use of surfactant in the delivery room.

Regarding neonatal evolution, the variables analyzed were: respiratory distress syndrome (RDS) with clinical and radiological diagnosis; ventilatory assistance time (continuous positive airway pressure - nasal CPAP and/or mechanical ventilation); early (<72 h life) or late (> 72 h) sepsis; brain alterations diagnosed by brain ultrasonography; diagnosis of bronchopulmonary dysplasia (BPD) defined by the need for supplemental oxygen at 36 weeks of postmenstrual age; death or hospital discharge. Diagnosis of necrotizing enterocolitis was defined by the presence of at least one clinical sign: bilious gastric aspirate or vomiting; abdominal distension; either occult or evident blood in the stool without anal fissure and the presence of at least one radiological sign included intestinal pneumatosis, gas in the hepatobiliary system and/or pneumoperitoneum.

Brain ultrasonography was performed on all newborns who survived up to 7 days. In newborns who had more than one examination, the worst ultrasound finding was considered. Cystic leukomalacia was defined by the presence of small cavities close to the lateral ventricles, and peri-intraventricular hemorrhage was classified according to the criteria of Papile et al. [18] and considered severe when it reached grade III or IV. The examinations were performed with GE® equipment by two neonatologists who specialized in diagnostic imaging, responsible for the routine assessment of preterm infants younger than 34 weeks of gestation.

Data analysis after completeness was checked, data were coded and entered Microsoft Excel and exported to statistical software SPSS version 22 for analysis. We used descriptive statistics to describe parameters collected from the files. Bivariate and multiple logistic regression were done to determine factors associated with unfavorable perinatal outcome. Variables which did not show statistical significance in the bivariate analysis were excluded from the multivariate analysis. P-value less than 0.05 and 95% confidence interval not including 1 were considered statically significant.

Ethical consideration

Ethical approval was by the Research Ethics Committee by the Foundation and Teaching and Research in Health Sciences - FEPECS, of the Health Department of the Federal District under registration CAAE 58280716.9.0000.5553, approval number

2,137,741. Regarding informed consent, the ethics committee waived the requirement for informed consent; however, confidentiality was maintained.

Results

In the period from January 1, 2014, to July 31, 2019, 22,441 babies with gestational age of less than 37 weeks were born in the Federal District, with 1480 newborns admitted to the Neonatal ICU of the HMIB during this period. Of these, 695 met the inclusion criteria for the study. A total of 785 newborns (NB) were excluded for the following reasons: multiple malformations (85); gestational diabetes (206); transferred (108) and pregnant women with inconclusive data (154) and incomplete data (232). Of the 695 newborns included in the study, 308 were born to hypertensive mothers (G1), and 387 were born to normotensive mothers (G2).

The distribution of hypertensive disease in G1 was present in 21.1% (151 pregnant women), pre-eclampsia in 6.9 % (48 pregnant women), 4.5% (31 pregnant women), eclampsia in 7.3% (51 pregnant women) chronic arterial hypertension alone and superimposed on pre-eclampsia and chronic hypertension in 0.6 % (4 pregnant women) and HEELP Syndrome 3.2% (22 pregnant women).

Table 1 describes the clinical characteristics presented by the women studied in G1 and G2. There was no difference between groups regarding gestational age and the use of antenatal corticosteroids. The use of magnesium sulfate was frequent in G1, and cesarean delivery was more common in this group.

Table 2 shows the morbidity of the newborns studied. The anthropometric measurements of birth weight and head circumference were significantly lower in G1 and had a higher risk of SGI NB (OR 2.4; CI 95% 1.6 – 3.6; p <0.00), as well as a greater risk of being born with a weight less than 850 g (OR 2.4; 95% CI 1.4 – 3.5; p <0.00). Newborns in group G1 had more necrotizing enterocolitis (OR 2.0; CI 95% 1.1– 3.7; p<0.03), but resuscitation in the delivery room and the need to use surfactant and oxygen dependence at 36 weeks of gestational age did not differ between groups. However, the G1 stay more time on mechanical ventilation (Table 2).

The changes in brain ultrasound until the first week of life, as well as the worst ultrasound image described before discharge or death, did not differ between groups (Table 3).

Being born in group G2 was a protective factor against death during the hospitalization period (OR 0.7; 95% CI 0.5 - 0.9; p <0.01), however, no difference was

observed at 18 months of chronological age, with rates of 95.3% in G1 and 92.1% in G2. Exclusive breastfeeding at discharge was 73.6% in the group of hypertensive women and 77.7% in the group of normotensive mothers. There were no significant differences between groups (Table 3).

Discussion

In a classic study, Villar et al. [19] compared the perinatal results in the following subgroups: pre-eclampsia and SGA, gestational hypertension and SGA, and SGA not explained as a reference group. Women with pre-eclampsia and SGA had a higher risk of their newborns staying in the neonatal ICU and experiencing neonatal death after adjusting the study for the location and socioeconomic status, but this difference disappeared after adjusting for birth weight and gestational age. This suggests that the excess risk associated with pre-eclampsia and SGA can be explained by these variables [19].

It was observed that most women attended prenatal care; however, normotensive mothers had fewer consultations (5.3 ± 2.4 and 5.0 ± 2.4, p = 0.63). Adequate monitoring of the pregnant woman is extremely important in preventing both maternal and fetal morbidity and mortality. Webster et al. [20], in a systematic review with meta-analysis, suggest that antihypertensive treatment reduces the risk of severe hypertension in pregnant women with chronic hypertension and, consequently, fewer maternal and neonatal complications. However, the lack of randomized controlled trials does not clarify which is the best therapeutic option [20].

Hypertensive pregnant women were older, with a mean age of 28.2 ± 6.8 compared to normotensive women, 26.1 ± 6.9 (p = 0.00). The prevalence of gestational hypertensive disease increases proportionally with the increase in maternal age and occurs more commonly in women over 34 years old; the data collected by this study demonstrate the same trend [21,22].

The use of antenatal corticosteroids above 70% in both groups reflects obstetric care; the benefits of this therapy are described in the literature in aspects such as decreased risk of neonatal death, the occurrence of respiratory distress syndrome and intraventricular hemorrhage [21-22].

Magnesium sulfate has been well evaluated as an effective intervention in the treatment of women with preeclampsia, ~~became~~ the drug of choice for the peripartum care of women with pre-eclampsia and eclampsia [23-25]. It was observed that in the studied

group, 59.7% of hypertensive pregnant women used magnesium sulfate, but only 36.4% of normotensive pregnant women. The data presented show that obstetric team needs to be made aware of the need to use magnesium sulfate in all pregnant women at risk of premature birth [23-25].

The preferred mode of delivery among hypertensive pregnant women was cesarean delivery in 68.5% of cases (OR 0.2; 95% CI 0.2 – 0.3), reflecting the need to use this mode due to maternal and/or fetal risk. Due to the severity of the condition, cesarean section has long been the preferred mode of delivery in cases of hypertensive syndrome. However, the American College of Obstetrics and Gynecology (ACOG) recommends vaginal delivery for this group of pregnant women [26]. It is suggested that vaginal delivery is safer than operative delivery in women with pre-eclampsia and should be attempted. The isolated diagnosis of pre-eclampsia should not be seen as an immediate indication for cesarean delivery; however, hypertensive pregnancy disease may be accompanied by other problems such as fetal distress and anomalous presentations, which constitute an indication for cesarean section [26].

Chorioaminionitis were more frequent in G2, presenting 25.3%, and 13.0% in G1 ($p < 0.00$). We can consider that sudden infectious conditions led to premature birth in newborns (NB) in the control group of this study. Although the causes of approximately half the cases of prematurity are of unknown etiology, infectious conditions are classically described as risk factors for prematurity [27]

The urinary infection was also more frequent in G2 23.0% and only in 12.0% G1. Gagliardi L et al [28] define the etiological background of very/extremely preterm birth can be divided into two main categories: intrauterine infection/inflammation and placental vascular dysfunction. The first category is associated with chorioamnionitis (CA), preterm labor, premature rupture of membranes (PROM), placental abruption, and cervical insufficiency, whereas the second category is associated with gestational hypertensive disorders and condition known as fetal indication/fetal growth restriction [28].

In this study it is possible to observe that this condition of severity is expressed as a serious problem within neonatal units' maternal hypertension and infection. This study shows that the group born of hypertensive disease was present in 21.1%, pre-eclampsia in 6.9 %, 4.5%, eclampsia in 7.3% chronic arterial hypertension alone and superimposed on pre-eclampsia and chronic hypertension in 0.6 % and HEELP Syndrome 3.2%.

Studies have pointed to the high risk of adverse outcomes in pregnant women who have overlapping pathologies [3-5]. It was not possible in this study to consider the subtypes diagnosed in the different groups under a more detailed analysis, as the choice was made not to stratify the samples for an individualized analysis of the cases.

However, it was observed that the newborns born to hypertensive mothers had lower birth weight (1054.2 ± 377.3 grams) compared to controls (1321.8 ± 352.5 grams p = 0.00). The same occurred with head circumference (27.8 ±4.2 cm in G1 and 28.8 cm ± 3.2 in G2, p <0.00). In addition, infants born to hypertensive mothers had a higher frequency of intrauterine growth restriction, with 23.7% being classified as small for gestational age (SGA), compared to the control group, at 10.1% (OR 2.4; 95% CI 1.6 – 3.6). These findings are in accordance with studies that found the same association between maternal arterial hypertension and NB with growth restriction [5,12,28].

The weight at discharge, showed an asymmetrical distribution. In this context, the median observed in G1 1970 grams and G2 1980 grams with p = 0.75; however, there was no significant difference between both groups.

When evaluating the babies again, this time at 18 months of age, an attempt was made to identify whether gestational hypertensive disease would be a risk factor for growth disorders in low-weight preterm infants. For this, logistic regression models were constructed, controlled by gestational age and sex, including maternal hypertension. In the classification between birth weight and gestational age, it was observed that HDP was not a risk factor for inadequate weight increase (OR = 0.54; 95% CI: 0.21 - 2.1) at 18 months of corrected age. Similar data were observed by Kiy et al. [28], in a cohort of premature infants at 18 months.

It was identified in the present study that gestational arterial hypertension increased the chance of ~~small NB for gestational age (~~SGA~~)~~ by approximately two and a half times (OR = 2.4; 95% CI: 1.6 – 3.6).

Considering this was a retrospective study, there was a difficulty in finding adequate records of height. The ordering of the main results of this study alerts us to the higher frequency of growth disorder in low-birth-weight preterm children born to hypertensive mothers [8,10,11].

There were no differences regarding the need for resuscitation and the diagnosis of respiratory distress syndrome, or the need for exogenous surfactant. Although a significantly increased risk for RDS was not found, some authors suggest that this type of association is possible [10,11].

Maternal hypertension and prematurity can lead to immaturity in the fetal gastrointestinal tract, poor vascular supply, and alteration of the intestinal microbiota, which in turn can be associated with a cascade of events, culminating in the development of necrotizing enterocolitis (NEC) [29,30]. In the present study, a significant association was observed between hypertensive maternal disease and neonatal NEC OR of 3.8 (95% CI 1.7 - 8.6),

There were no differences between the groups regarding the ~~time~~ of use of mechanical ventilation, nasal CPAP and oxygen at 28 days or 36 weeks of gestational age, demonstrating that respiratory changes are not linked to maternal pressure changes, but to the complications and limitations of prematurity and low birth weight.

The literature is conflicting about the effects of pre-eclampsia and the development of retinopathy of prematurity (ROP), one of the main causes of childhood blindness worldwide. No differences in the diagnosis of ROP between groups were observed in this study [32]. Retinopathy of prematurity is initially manifested with delayed physiological vascular development of the retina, followed by aberrant vasoproliferation, and is highly correlated with extreme prematurity and low postnatal growth. [32,33].

In the studied sample, there were no differences between groups regarding the presence of cerebral hemorrhage, however we observed that leukomalacia was more frequent in G1 OR 3.1 CI 95% 1.1 - 8.8. These changes can be identified by brain ultrasound and are associated with an increased risk of neurodevelopmental disorders, which may inform the need for greater pediatric surveillance of babies exposed to maternal hypertensive disease. Ensuring early stimulation can help improve the outcome of neurological development; therefore, the neurological prognosis for these premature babies whose mothers have high blood pressure is still very controversial [10,12].

Although the diagnosis of early neonatal sepsis is more frequent in G2, no statistical difference was observed between groups OR 0.8 of CI 95% 0.6 - 1.1; the same was found in the diagnosis of late sepsis, which was more frequent in G1, but without statistical difference OR 1.3 of CI 95% 1.0 - 1.8.

Higher neonatal mortality was observed in the group of hypertensive mothers, confirming the results of previous studies [8,10,11,34,35]. When the results were adjusted for birth weight, the presence of hypertension was not shown to be an isolated risk factor for death (OR 1.3 of 95% CI 0.8 - 1.9). This result follows what has been described in the literature.

There are important limitations of this study that must be considered when interpreting the reported findings. As the project was of a retrospective nature, suggesting that other confounding factors may be operating and have not been identified. However, it was still possible to detect significant and clinically relevant differences between preterm infants born to hypertensive mothers compared to normotensive mothers.

The results of this study show that gestational hypertensive disorder has short-term repercussions in premature newborns, who are born more frequently by cesarean section and are at greater risk of being born small for gestational age and of presenting necrotizing enterocolitis. Studies with a larger sample size of a multicentric and prospective nature with late follow-up are necessary to consolidate these findings.

In these NB studies, it is possible to see that the population of newborns with Hypertensive Disorders of Pregnancy treated at the HMIB had more mortality and morbidity. We recommend careful monitoring of hypertensive pregnant women with a multidisciplinary and careful approach, guiding the follow-up of these women and their children to improve the clinical results of these newborns.

Acknowledgements

We gratefully appreciate the support of medical doctors in the Neonatal Intensive Care Unit of the Brasília Mother and Child Hospital.

Conflict of interest

The authors declare that they have no conflict of interests.

Author contributions

All authors were involved in the concept and design of the study. PRM and MRCGN conducted independent literature searches and quality assessment; MDRM performed data extraction and wrote the first draft of the manuscript. KN and PRM supervised the article selection, quality assessment and data presentation.

All authors have read and approved the final manuscript and agree to be accountable for all aspects of the work. No form of payment was given to anyone to produce the manuscript.

Funding information

Not applicable.

References

1. Shah S, Gupta A. Hypertensive disorders of pregnancy. *Cardiol Clin* 2019; **37**: 345–354.
2. Umesawa M, Kobashi G. Epidemiology of hypertensive disorders in pregnancy: prevalence, risk factors, predictors and prognosis. *Hypertens Res* 2017; **40**: 213–220.
3. Brown MA, Magee LA, Kenny LC, Karumanchi SA, McCarthy FP, Saito S, *et al.* The hypertensive disorders of pregnancy: ISSHP classification, diagnosis & management recommendations for international practice. *Pregnancy Hypertens* 2018; **13**: 291–310.
4. Yu H, He Y, Mao Z, Dong W, Fu X, Lei X. Hypertensive disorders during pregnancy and elevated blood pressure in the offspring: a systematical review and meta-analysis protocol. *Medicine* 2019; **98**: e15677.
5. Nzelu D, Dumitrascu-Biris D, Kay P, Nicolaides KH, Kametas NA. Severe hypertension, preeclampsia and small for gestational age in women with chronic hypertension diagnosed before and during pregnancy. *Pregnancy Hypertens* 2018; **14**: 200–204.
6. Rodrigues Â, Barata C, Marques I, Almeida MC. Diagnosis of white coat hypertension and pregnancy outcomes. *Pregnancy Hypertens* 2018; **14**: 121–124.
7. Wang Y, Hao M, Sampson S, Xia J. Elective delivery versus expectant management for pre-eclampsia: a meta-analysis of RCTs. *Arch Gynecol Obstet* 2017; **295**: 607–622.
8. Omani-Samani R, Ranjbaran M, Amini P, Esmailzadeh A, Sepidarkish M, Almasi-Hashiani A. Adverse maternal and neonatal outcomes in women with preeclampsia in Iran. *J Matern Fetal Neonatal Med* 2019; **32**: 212–216.
9. Nisa MU, Anjum S. Elective induction versus expectant management of mild pre-eclampsia at term. *J Coll Physicians Surg Pak* 2018; **28**: 677–680.
10. Marins LR, Anizelli LB, Romanowski MD, Sarquis AL. How does preeclampsia affect neonates? Highlights in the disease's immunity. *J Matern Fetal Neonatal Med* 2019; **32**: 1205–1212.

11. O'Gorman N, Nicolaides KH, Poon LC. The use of ultrasound and other markers for early detection of preeclampsia. Womens Health (Lond). 2016;**12**(2):199-207.

12. Maher GM, O'Keeffe GW, Kearney PM, Kenny LC, Dinan TG, Mattsson M, *et al.* Association of hypertensive disorders of pregnancy with risk of neurodevelopmental disorders in offspring: a systematic review and meta-analysis. *JAMA Psychiatry* 2018; **75**: 809–819

13. Nathan HL, Seed PT, Hezelgrave NL, De Greeff A, Lawley E, Conti-Ramsden F, *et al.* Maternal and perinatal adverse outcomes in women with pre-eclampsia cared for at facility-level in South Africa: a prospective cohort study. *J Glob Health* 2018; **8**: 020401.

14. von Elm E, Altman DG, Egger M, Pocock SJ, Gotzsche PC, Vandenbroucke JP, *et al.* The strengthening the reporting of observational studies in epidemiology (STROBE) statement: guidelines for reporting observational studies. *J Clin Epidemiol* 2008; **61**: 344–349.

15. Ballard JL, Khoury JC, Wedig K, Wang L, Eilers-Walsman BL, Lipp R. New Ballard Score, expanded to include extremely premature infants. *J Pediatr*. 1991;**119**: 417-423.

16. Lubchenco L, Hansman C, Dressler M, Boyd E. Intrauterine growth as estimated from liveborn birth-weight data at 24 to 42 weeks of gestation. *Pediatrics* 1963; **32**: 793–800.

17. Margotto PR. Curvas de crescimento intra-uterino: estudo de 4413 recém-nascidos únicos de gestações normais. *J Pediatr* 1995; **71**: 11–21.

18. Papile LA, Burstein J, Burstein R, Koffler H. Incidence and evolution of subependymal and intraventricular hemorrhage: A study of infants with birth weights less than 1,500 gm. *J Pediatr* 1978; **92**: 529–534.

19. Villar J, Carroli G, Wojdyla D, Abalos E, Giordano D, Ba'aqeel H, *et al.* Preeclampsia, gestational hypertension and intrauterine growth restriction, related or independent conditions? *Am J Obstet Gynecol* 2006; **194**: 921–931.

20. Webster LM, Conti-Ramsden F, Seed PT, Webb AJ, Nelson-Piercy C, Chappell LC. Impact of antihypertensive treatment on maternal and perinatal outcomes in pregnancy complicated by chronic hypertension: a systematic review and meta-analysis. *J Am Heart Assoc* 2017; **6**: e005526.

21. Shen M, Smith GN, Rodger M, White RR, Walker MC, Wen SW. Comparison of risk factors and outcomes of gestational hypertension and pre-eclampsia. *PLoS One* 2017; **12**: e0175914.

22. Deshmukh M, Patole S. Antenatal corticosteroids for neonates born before 25 weeks-a systematic review and meta-analysis. *PLoS One* 2017; **12**: e0176090.

23. Duley L, Gülmezoglu AM, Henderson-Smart DJ, Duley L. Magnesium sulphate and other anticonvulsants for women with pre-eclampsia. *Cochrane Database Syst Rev* 2010; **2010**: CD000025.

24. Jameson RA, Bernstein HB. Magnesium sulfate and novel therapies to promote neuroprotection. *Clin Perinatol* 2019; **46**: 187–201.

25. American College of Obstetricians Gynecologists, Task Force on Hypertension in Pregnancy. Hypertension in pregnancy. Report of the American college of obstetricians and gynecologists' task force on hypertension in pregnancy. *Obstet Gynecol* 2013; **122**: 1122–1131.

26. Vogel JP, Chawanpaiboon S, Moller A-B, Watananirun K, Bonet M, Lumbiganon P. The global epidemiology of preterm birth. *Best Pract Res Clin Obstet Gynaecol* 2018; **52**: 3–12.

27. Razak A, Florendo-Chin A, Banfield L, Abdul Wahab MG, McDonald S, Shah PS, *et al.* Pregnancy-induced hypertension and neonatal outcomes: a systematic review and meta-analysis. *J Perinatol* 2018; **38**: 46–53.

28. Gagliardi L, Rusconi F, Bellù R, Zanini R; Italian Neonatal Network. Association of maternal hypertension and chorioamnionitis with preterm outcomes. *Pediatrics*. 2014; **134**:e154-e161.

29. Kiy AM, Rugolo LMSS, Luca AKCD, Corrente JE. Growth of preterm low birth weight infants until 24 months corrected age: effect of maternal hypertension. *J Pediatr* 2015; **91**: 256–262.

29. Yang C-C, Tang P-L, Liu P-Y, Huang W-C, Chen Y-Y, Wang H-P, *et al.* Maternal pregnancy-induced hypertension increases subsequent neonatal necrotizing enterocolitis risk: a nationwide population-based retrospective cohort study in Taiwan. *Medicine* 2018; **97**: e11739.

30. Neu J. Necrotizing enterocolitis. *World Rev Nutr Diet* 2014; **110**: 253–263.

31. Vieira AA, David BBL, Lino RRG, Duarte LDB, Bueno AC. Avaliação dos fatores perinatais que interferem na incidência de enterocolite necrosante em

recém-nascidos de muito baixo peso. *Rev Bras Ginecol Obstet* 2013; **35**: 363–367.

32. Zhu T, Zhang L, Zhao F, Qu Y, Mu D. Association of maternal hypertensive disorders with retinopathy of prematurity: a systematic review and meta-analysis. *PLoS One* 2017; **12**: e0175374.

33. Bashinsky AL. Retinopathy of prematurity. *N C Med J* 2017; **78**: 124–128.

34. Ndwiga C, Odwe G, Pooja S, Ogutu O, Osoti A, E Warren C. Clinical presentation and outcomes of pre-eclampsia and eclampsia at a national hospital, Kenya: A retrospective cohort study. *PLoS One*. 2020;**15**:e0233323.

35. Dassah ET, Kusi-Mensah E, Morhe ESK, Odoi AT. Maternal and perinatal outcomes among women with hypertensive disorders in pregnancy in Kumasi, Ghana. *PLoS One*. 2019;**14**: e0223478.

Table 1. Characteristics of the studied pregnant women divided considering the presence or not of Hypertensive disorders of pregnancy.

	G1<br>n: 308 NB	G2<br>n: 387 NB	p	OR<br>(95% CI)
Maternal Age (years)	28.2 ± 6.8	26.1 ± 6.9	0.00	-
Number of prenatal consultations	5.3 ± 2.4	5. ± 2.4	0.63	-
Gestational Age	28.9± 2.9	29.8 ± 2.2	0.00	-
Cesarean delivery%	68.5	34.4	0.00	0.2 (0.2 to 0.3)
Urinary infection %	12.0	23.0	0.00	0.4 (0.3 to 0.7)
Chorioamnionitis%	13.0	25.3	0.00	0.4 (0.3 to 0.6)
Prenatal corticosteroids%	77.3	73.1	0.21	1.2 (0.8 to 1.8)
Magnesium Sulfate%	59.7	36.4	0.00	2.6 (1.9 to 3.5)

Data: mean ± SD, p-Value < 0,05 via Student't – test; Data: n (percent), p – Value < 0,05 via X^2 test

Table 2. Neonates' morbidity considering the presence or not of Hypertensive disorders of pregnancy.

	G1<br>n: 308 NB	G2<br>n: 387 NB	p	OR<br>(95% CI)
Birth weight (grams)	1054.2± 377.3	1321.8 ± 352.5	0.00	-
Head circumference (cm)	27.8 ± 4.2	28.8 ± 3.2	0.00	-
Mechanical ventilation (days)	7.3 ± 15.6	5.2 ± 13.0	0.05	-
Apgar 1 min	5.9 ± 2.1	6.3 ± 2.1	0.02	-
Apgar 5 min	7.8 ± 1.6	7.8 ± 1.5	0.97	-
Hospitalization time in days	37.8 ± 32.3	36.7 ± 39.5	0.76	-
Weight at hospital discharge	1919.5 ± 226.9	1.811.9 ± 346.1	0.00	-
NB <850 g %	23.7	11.4	0.00	2.4 (1.4 to 3.5)
SGA rating%	23.7	10.1	0.00	2.4 (1.6 to 3.6)
Male %	50.3	48.8	0.70	1.0 (0.8 to 1.4)
Neonatal resuscitation %	66.6	61.5	0.17	1.2 (0.9 to 1.7)
Use of surfactant%	47.7	41.6	0.10	1.3 (0.9 to 1.7)
Early sepsis%	34,1	39.3	0.17	0.8 (0.6 to 1.1)
Later sepsis%	37.7	31.0	0.07	1.3 (1.0 to 1.8)
Use of O2 at 36 weeks%	23.0	24.4	0.81	0.9 (0.6 to 1.5)
Necrotizing Enterocolitis%	8.4	4.4	0.03	2.0 (1.1 to 3.7)
Retinopathy of prematurity%	13.6	15.0	0,63	0.8 (0.5 to 1.4)
Leukomalacia (%)	6.3	2.1	0.03	3.1 (1.1 to 8.8)

HIV: Intraventricular Hemorrhage, Data: n (percent), p – Value< 0,05 via X^2 test
Data: mean ± SD, p-Value < 0,05 via Student't – test; NB – New Born; SGA - Small for gestational age.

Table 3. Survival at discharge and at 18 months in neonates considering the presence or not of Hypertensive disorders of pregnancy.

	G1<br>n: 308 RN	G2<br>n: 387 RN	p	OR (IC 95%)	
Survival at discharge (%)	69.8	77.3	0.03	0.7	(0.5 to 0.9)
Exclusive breastfeeding at discharge (%)	73.4	77.7	0.25	0.8	(0.5 to 1.1)
Survival at 18 months (%)	95.3	92.1	0.15	1.7	(0.9 to 3.7)
Breastfeeding at 18 months (%)	66.7	70.9	0.28	0.8	(0.6 to 1.1)
Weight at 18 months (kg)	8.868 ± 0.720	8.830 ± 0.670	0.72		

Data: mean ± SD, p-Value < 0,05 via Student't – test / Data: n (percent), p – Value< 0,05 via X^2 test

Figure Click here to access/download;Figure;Figura 1 revisada.docx

Figure 1. Schematic diagram showing the number of participants medical birth registry, 2014–2019.

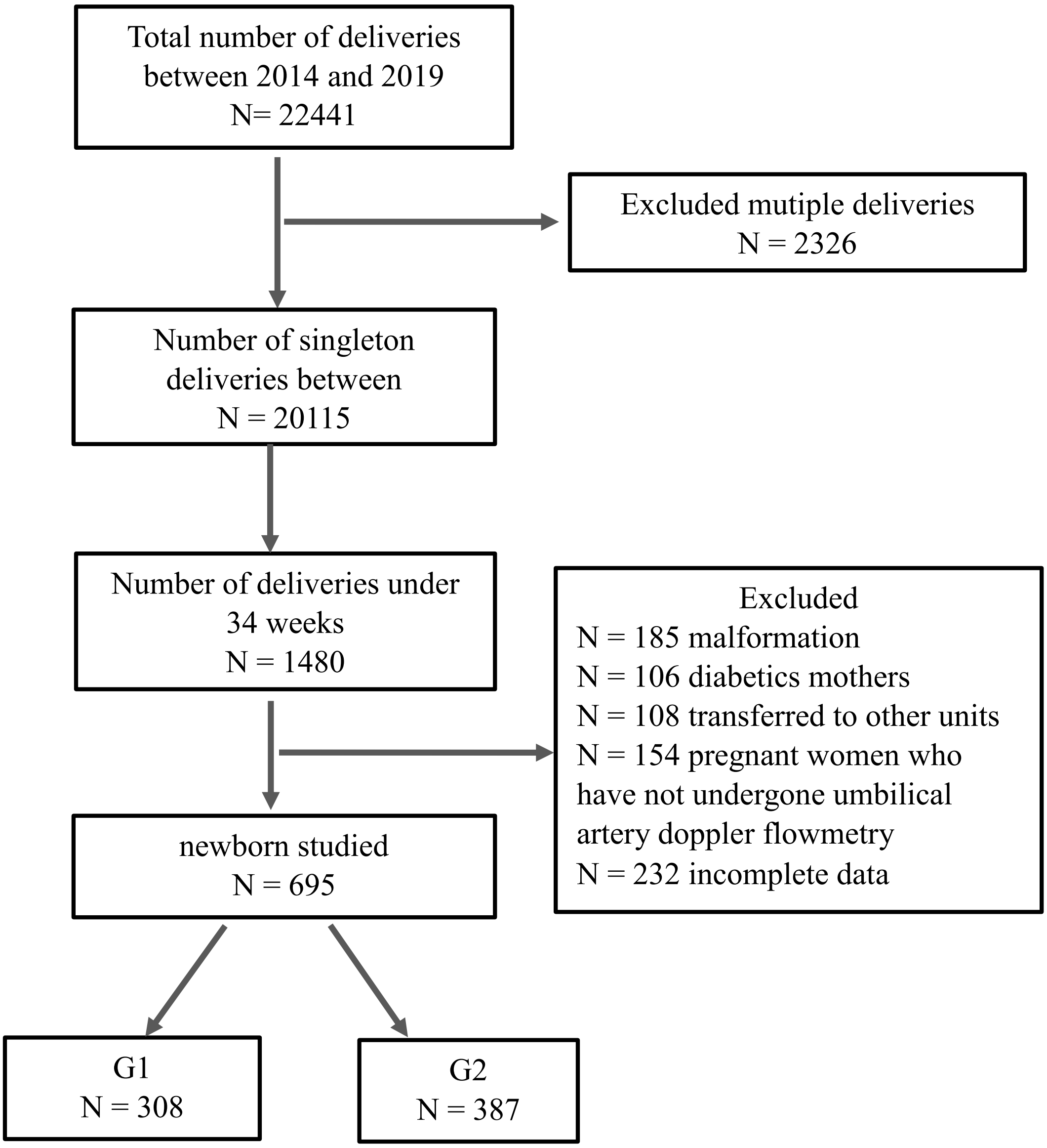

Hypertension induced by pregnancy and neonatal outcome: Results from a retrospective cohort study in preterm under 34 weeks.

Commented [MN1]: Given the recommendation added to the title premature

Moura, MDR [1*] , Margotto P.R.[2], Costa K.N.[3], Novaes M.R.C.G.[4]
Marta David Rocha de Moura [1]
Paulo Roberto Margotto [2]
Karina Nascimento Costa [3]
Maria Rita Carvalho Garbi Novaes [4]

[1] Assistant doctor, neonatal intensive care unit, Brasília Mother and Child Hospital , SGAS 608 A, Asa Sul , Brasília – DF , 70603900.

[2] Neonatal Senior doctor, intensive care unit, Brasília Mother and Child Hospital , SGAS 608 A, Asa Sul , Brasília – DF , 70603900.

[3] Department of Child and Adolescent Medicine, University of Brasília, **UnB** - Brasília, DF, 70910-900.

[4] Higher School of Health Sciences, SMHN Conjunto A B 01 FEPECS - Asa Norte, Brasília - DF, 70710-907.

* Corresponding author: Marta David Rocha de Moura, email: marta.rocha@escs.edu.br.

[¶] These authors contributed equally to this work.

[&] these authors also contributed equally to this work.

Abstract

Commented [MN2]: We make changes to be more clean

Objective: The present study seeks to assess the impact of gestational hypertensive disorders on premature newborns below 34 weeks and to establish the main morbidities and mortality in the neonatal period and at 18 months.

Materials and methods: A retrospective observational study was carried out with 695 premature newborns of gestational age (GA) between 24 and 33 weeks and 6 days, born alive in the Neonatal ICU of Brasília's Mother and Child Hospital (HMIB), in the period from January 1, 2014, to July 31, 2019. In total, 308 infants were born to hypertensive

mothers (G1) and 387 to normotensive mothers (G2). Twin pregnancies and diabetic patients with severe malformations were excluded. Outcomes during hospitalization and outcomes of interest were evaluated: respiratory distress syndrome (RDS), brain ultrasonography, diagnosis of bronchopulmonary dysplasia (BPD), diagnosis of necrotizing enterocolitis, retinopathy of prematurity, breastfeeding rate at discharge, survival at discharge and at 18 months of chronological age and relationship between weight and gestational age.

Results: Newborns with hypertensive mothers had significantly lower measurements of birth weight and head circumference. The G1 group had a higher risk of small babies for gestational age Small for gestational age (OR 2.4; CI 95% 1.6 – 3.6; p <0.00), as well as a greater risk of being born with a weight less than 850 g (OR 2.4; 95% CI 1.2 – 3.5; p <0.00). Newborns of mothers with hypertension presented more necrotizing enterocolitis (OR 2.0; CI 95% 1.1-3.7); however, resuscitation in the delivery room and the need to use surfactant did not differ between groups, nor did the length of stay on mechanical ventilation, or dependence on oxygen at 36 weeks of gestational age. Survival was better in newborns of normotensive mothers, and this was a protective factor against death (OR 0.7; 95% CI 0.5 - 0.9; p <0.01). In the follow-up clinic, survival at 18 months of chronological age was similar between groups, with rates of 95.3% and 92.1% among hypertensive and normotensive mothers, respectively. Exclusive breastfeeding at discharge was 73.4% in the group of hypertensive women and 77.3% in the group of normotensive mothers. There were no significant differences between groups.

Conclusion: Among the analyzed outcomes, arterial hypertension during pregnancy can increase the risk of low weight, small babies for gestational age (SGA), deaths in the neonatal period and enterocolitis, with no differences in weight and survival at 18 months of chronological age. Arterial hypertension presents a high risk of prematurity in the neonatal period, with no difference at 18 months of age.

Introduction

Commented [MN3]: the previous paragraph referring to the types of hypertensive disorders in pregnancy was excluded

Hypertensive Disorders of Pregnancy (HDP) present a serious complication that affects approximately 2.5 to 3.0 percent of women, increasing the risk of maternal and neonatal complications [1,2]. Worldwide, hypertensive disorders remain the leading cause of maternal mortality related to pregnancy. [1]

Commented [MN4]: the term hypertensive syndrome has been replaced by hypertensive disease, this article was initially written in Portuguese and some translated terms have created confusion in the English language. All text was proofread by an English-speaking proofreader

Hypertensive disorders of pregnancy appear as a hypertensive condition that develops at any time after 20 weeks of pregnancy, accompanied or not by proteinuria. Among the manifestations of these syndromes is eclampsia, which presents with a convulsive component, and HELPP syndrome, which manifests with the presence of hemolysis, elevated liver enzymes and thrombocytopenia; HELLP is a severe form of preeclampsia and not a separate disorder [3,4].

Hypertensive Disorders of Pregnancy cannot be prevented; therefore, the identification of maternal risk factors becomes an important obstetric mission [1]. The risk factors are associated with the development of gestational hypertension, previous history of pre-eclampsia, primiparity, obesity, family history of pre-eclampsia, number of previous pregnancies (if any) and chronic medical conditions, such as hypertension and diabetes, maternal age greater than or equal to 40 years, obesity, diabetes mellitus, chronic kidney disease, systemic lupus erythematosus, presence of antiphospholipid, multiple pregnancies and high altitude. In addition, it is important to highlight the action of kidney transplant of live kidneys, which can increase the risk of developing pre-eclampsia in the recipients by up to six-fold more than in non-transplanted women [1-6].

The decision to terminate pregnancy must balance maternal and neonatal risks; thus, the basic objective of obstetrics is to carefully prolong pregnancy to improve the perinatal outcome without compromising maternal safety [7,8]. Some authors suggest that expectant control of pregnancy is associated with an increase in maternal complications in a pregnancy of 32 weeks or more [7-9].

The neonatal complications described here range from prematurity to fetal growth restriction. The latter is the most frequent neonatal complication in newborns (NB) with hypertensive mothers [10,12,13,14]. Perinatal mortality rates in growth-restricted neonates are 6 to 10 times that of those with normal growth [2,5]. Doppler ultrasonography in fetuses of hypertensive mothers is a way of assessing the severity of intrauterine growth restriction and intrauterine monitoring allows the disease progression to be observed non-invasively. [11].

Other morbidities described in newborns of mothers with hypertensive conditions are bronchopulmonary dysplasia (BPD), retinopathy of prematurity (ROP), sepsis and longer time on mechanical ventilation. Hematological changes such as thrombocytopenia and leukopenia are also frequently described in the literature [10].

Exposure to HDP may be associated with an increased risk of autism spectrum disorder (ASD) and attention deficit/hyperactivity disorder [12,13]. These findings

highlight the need for greater pediatric surveillance of newborns exposed to HDP to allow early interventions that can improve neurological development outcomes.

The aim of this study is to assess the impact of gestational hypertensive disorders on premature newborns below 34 weeks and to identify the mortality rate and morbidities at discharge and at 18 months of corrected gestational age.

Material and methods

Commented [MN5]: The methodology has been rewritten to meet the editorial guidelines.

Study design, setting and population.

Commented [MN6]: An adjustment and separation was made in the materials and methods to clarify the steps developed

This retrospective cohort study included preterm infants, with gestational age (GA) between 24 and 33 weeks and 6 days, born alive in a maternity center in Brasília, Federal District, Brazil, at the Mother and Child Hospital (Hospital Materno Infantil de Brasília, HMIB), and admitted to the Neonatal Intensive Care Unit of this Hospital, from January 1, 2014, to July 31, 2019.

HMIB is a Teaching and Referral Hospital in Federal District. The hospital This hospital was opened in1964 and it is a referral center since 1988 for neonates, women, and surgical children as tertiary hospital. Having a medical residency program in the areas of pediatrics, neonatology, pediatric intensive care, obstetrics, fetal medicine and pediatric surgery

A total of 18768 deliveries in the institution during the specified period. Preterm born in the period was 1765 newborns. (Figure 1)

Exclusion criteria were premature infants transferred directly from the delivery room to another service, as well as deaths found in the delivery room, twins, newborns from other services and patients with severe malformations, diabetics mothers and NB that have not undergone umbilical artery doppler flowmetry. All patients included in the study were followed up during hospitalization and evaluated for the outcomes of interest at 28 days of life or at discharge/death/transfer and at 18 months of corrected gestational age.

Sample size calculations and statistical analysis

Commented [MN7]: all data were reviewed and reanalyzed by the statistical team. Some data has been removed or adjusted to provide more clarity to the study

In The study design followed the STROBE checklist recommendations of the Equator Network [15]. Sample size calculations and statistical analysis. Sample size calculations were done in Epi Info TM version 7.2.2.6 (Centers for Disease Control and Prevention (CDC); USA) at 80% power and 95% confidence interval. Based on a 10%

prevalence of hypertension during pregnancy, considering a 99.9% confidence level, 90% power and a 1:1 ratio (exposed and unexposed). The estimated sample size was 432 newborns, including 216 newborns with hypertensive mothers and 216 newborns with normotensive mothers. During the study period, it was possible to select 695 NBs for the study, divided into two groups, G1 - hypertensive pregnant women, with 308 newborns, and G2 - normotensive pregnant women with 387 newborns.

Operational definitions

Hypertension in pregnancy was defined as systolic blood pressure (BP) ≥140 mmHg and/or a diastolic BP ≥ 90 mmHg. Preeclampsia was defined as characterized by a BP of 140/90 mm Hg or greater after 20 weeks' gestation in a woman with previously normal BP and who have proteinuria. Eclampsia was defined as seizures that cannot be attributable to other causes, in a woman with preeclampsia. In this study, hypertensive disorders of pregnancy were classified as preeclampsia, gestational hypertension, chronic hypertension and preeclampsia superimposed on chronic hypertension. [3]

Data variables for the study order to assess the fetal outcomes of hypertensive disorders of pregnancy, data was obtained from the medical records of the study subjects. analyzed included the following gestational data, obtained from obstetric notes and confirmed in the postpartum period: prenatal care (> 2 consultations for premature births); maternal age; maternal infection (clinical chorioamnionitis or peripartum urinary tract infection); antenatal steroid use (> one dose administered before delivery, regardless of the type of steroid); use of magnesium sulfate, diagnosis of hypertensive disease of pregnancy (including variations of pre-eclampsia, eclampsia, HELLP syndrome and pre-eclampsia overlapping with chronic arterial hypertension); and the presence of Absent end-diastolic velocity waveform in gestational ultrasound up to 72 hours before and during delivery.

The data related to birth were gestational age defined by the best obstetric estimate, preferably the precise date of the last menstruation, followed by early obstetric ultrasound (up to the 18th week of pregnancy). In the absence of such data, gestational age (GA) was estimated by examining the newborn, performed immediately after birth or within 12 hours, using the method of New Ballard [15]. Other data analyzed were gender; birth weight; weight and gestational age classification to define small for gestational age (SGA), according to the criteria of Lubchenco et al. [16] and Margotto [17], from 29 weeks; Apgar of the 1st and 5th minutes; need for resuscitation in the delivery room

Commented [MN8]: The Margotto curve was made with newborns attended at the service and was validated by the Latin American Center for Perinatology (CLAP) in 1990 and is used throughout the federal district and several regions of Brazil in classification

defined using positive pressure ventilation under a mask; and the use of surfactant in the delivery room.

Commented [MN9]: Corrections in numbering of tables and identification of groups were made.

Regarding neonatal evolution, the variables analyzed were: respiratory distress syndrome (RDS) with clinical and radiological diagnosis; ventilatory assistance time (continuous positive airway pressure - nasal CPAP and/or mechanical ventilation); early (<72 h life) or late (> 72 h) sepsis; brain alterations diagnosed by brain ultrasonography; diagnosis of bronchopulmonary dysplasia (BPD) defined by the need for supplemental oxygen at 36 weeks of postmenstrual age; death or hospital discharge. Diagnosis of necrotizing enterocolitis was defined by the presence of at least one clinical sign: bilious gastric aspirate or vomiting; abdominal distension; either occult or evident blood in the stool without anal fissure and the presence of at least one radiological sign included intestinal pneumatosis, gas in the hepatobiliary system and/or pneumoperitoneum.

Brain ultrasonography was performed on all newborns who survived up to 7 days. In newborns who had more than one examination, the worst ultrasound finding was considered. Cystic leukomalacia was defined by the presence of small cavities close to the lateral ventricles, and peri-intraventricular hemorrhage was classified according to the criteria of Papile et al. [18] and considered severe when it reached grade III or IV. The examinations were performed with GE® equipment by two neonatologists who specialized in diagnostic imaging, responsible for the routine assessment of preterm infants younger than 34 weeks of gestation.

Commented [MN10]: All terms

Data analysis after completeness was checked, data were coded and entered Microsoft Excel and exported to statistical software SPSS version 22 for analysis. We used descriptive statistics to describe parameters collected from the files. Bivariate and multiple logistic regression were done to determine factors associated with unfavorable perinatal outcome. Variables which did not show statistical significance in the bivariate analysis were excluded from the multivariate analysis. P-value less than 0.05 and 95% confidence interval not including 1 were considered statically significant.

Ethical consideration

Ethical approval was by the Research Ethics Committee by the Foundation and Teaching and Research in Health Sciences - FEPECS, of the Health Department of the Federal District under registration CAAE 58280716.9.0000.5553, approval number

2,137,741. Regarding informed consent, the ethics committee waived the requirement for informed consent; however, confidentiality was maintained.

Results

In the period from January 1, 2014, to July 31, 2019, 22,441 babies with gestational age of less than 37 weeks were born in the Federal District, with 1480 newborns admitted to the Neonatal ICU of the HMIB during this period. Of these, 695 met the inclusion criteria for the study. A total of 785 newborns (NB) were excluded for the following reasons: multiple malformations (85); gestational diabetes (206); transferred (108) and pregnant women with inconclusive data (154) and incomplete data (232). Of the 695 newborns included in the study, 308 were born to hypertensive mothers (G1), and 387 were born to normotensive mothers (G2).

The distribution of hypertensive disease in G1 was present in 21.1% (151 pregnant women), pre-eclampsia in 6.9 % (48 pregnant women), 4.5% (31 pregnant women), eclampsia in 7.3% (51 pregnant women) chronic arterial hypertension alone and superimposed on pre-eclampsia and chronic hypertension in 0.6 % (4 pregnant women) and HEELP Syndrome 3.2% (22 pregnant women).

Table 1 describes the clinical characteristics presented by the women studied in G1 and G2. There was no difference between groups regarding gestational age and the use of antenatal corticosteroids. The use of magnesium sulfate was frequent in G1, and cesarean delivery was more common in this group.

Table 2 shows the morbidity of the newborns studied. The anthropometric measurements of birth weight and head circumference were significantly lower in G1 and had a higher risk of SGI NB (OR 2.4; CI 95% 1.6 – 3.6; p <0.00), as well as a greater risk of being born with a weight less than 850 g (OR 2.4; 95% CI 1.4 – 3.5; p <0.00). Newborns in group G1 had more necrotizing enterocolitis (OR 2.0; CI 95% 1.1– 3.7; p<0.03), but resuscitation in the delivery room and the need to use surfactant and oxygen dependence at 36 weeks of gestational age did not differ between groups. However, the G1 stay more time on mechanical ventilation (Table 2).

The changes in brain ultrasound until the first week of life, as well as the worst ultrasound image described before discharge or death, did not differ between groups (Table 3).

Being born in group G2 was a protective factor against death during the hospitalization period (OR 0.7; 95% CI 0.5 - 0.9; p <0.01), however, no difference was

Commented [MN11]: In compliance with the recommendations, paragraphs were deleted and reorganized to make the text more direct and objective.
thank you very much for the suggestions

observed at 18 months of chronological age, with rates of 95.3% in G1 and 92.1% in G2. Exclusive breastfeeding at discharge was 73.6% in the group of hypertensive women and 77.7% in the group of normotensive mothers. There were no significant differences between groups (Table 3).

Discussion

Commented [MN12]: In response to the reviewers' requests, the paragraph is rewritten. The paragraph suffers from magnesium sulfate.

In a classic study, Villar et al. [19] compared the perinatal results in the following subgroups: pre-eclampsia and SGA, gestational hypertension and SGA, and SGA not explained as a reference group. Women with pre-eclampsia and SGA had a higher risk of their newborns staying in the neonatal ICU and experiencing neonatal death after adjusting the study for the location and socioeconomic status, but this difference disappeared after adjusting for birth weight and gestational age. This suggests that the excess risk associated with pre-eclampsia and SGA can be explained by these variables [19].

It was observed that most women attended prenatal care; however, normotensive mothers had fewer consultations (5.3 ± 2.4 and 5.0 ± 2.4, p = 0.63). Adequate monitoring of the pregnant woman is extremely important in preventing both maternal and fetal morbidity and mortality. Webster et al. [20], in a systematic review with meta-analysis, suggest that antihypertensive treatment reduces the risk of severe hypertension in pregnant women with chronic hypertension and, consequently, fewer maternal and neonatal complications. However, the lack of randomized controlled trials does not clarify which is the best therapeutic option [20].

Hypertensive pregnant women were older, with a mean age of 28.2 ± 6.8 compared to normotensive women, 26.1 ± 6.9 (p = 0.00). The prevalence of gestational hypertensive disease increases proportionally with the increase in maternal age and occurs more commonly in women over 34 years old; the data collected by this study demonstrate the same trend [21,22].

The use of antenatal corticosteroids above 70% in both groups reflects obstetric care; the benefits of this therapy are described in the literature in aspects such as decreased risk of neonatal death, the occurrence of respiratory distress syndrome and intraventricular hemorrhage [21-22].

Magnesium sulfate has been well evaluated as an effective intervention in the treatment of women with preeclampsia, became the drug of choice for the peripartum care of women with pre-eclampsia and eclampsia [23-25]. It was observed that in the studied

group, 59.7% of hypertensive pregnant women used magnesium sulfate, but only 36.4% of normotensive pregnant women. The data presented show that obstetric team needs to be made aware of the need to use magnesium sulfate in all pregnant women at risk of premature birth [23-25].

The preferred mode of delivery among hypertensive pregnant women was cesarean delivery in 68.5% of cases (OR 0.2; 95% CI 0.2 – 0.3), reflecting the need to use this mode due to maternal and/or fetal risk. Due to the severity of the condition, cesarean section has long been the preferred mode of delivery in cases of hypertensive syndrome. However, the American College of Obstetrics and Gynecology (ACOG) recommends vaginal delivery for this group of pregnant women [26]. It is suggested that vaginal delivery is safer than operative delivery in women with pre-eclampsia and should be attempted. The isolated diagnosis of pre-eclampsia should not be seen as an immediate indication for cesarean delivery; however, hypertensive pregnancy disease may be accompanied by other problems such as fetal distress and anomalous presentations, which constitute an indication for cesarean section [26].

Chorioaminionitis were more frequent in G2, presenting 25.3%, and 13.0% in G1 (p <0.00). We can consider that sudden infectious conditions led to premature birth in newborns (NB) in the control group of this study. Although the causes of approximately half the cases of prematurity are of unknown etiology, infectious conditions are classically described as risk factors for prematurity [27]

The urinary infection was also more frequent in G2 23.0% and only in 12.0% G1. Gagliardi L et al [28] define the etiological background of very/extremely preterm birth can be divided into two main categories: intrauterine infection/inflammation and placental vascular dysfunction. The first category is associated with chorioamnionitis (CA), preterm labor, premature rupture of membranes (PROM), placental abruption, and cervical insufficiency, whereas the second category is associated with gestational hypertensive disorders and condition known as fetal indication/fetal growth restriction [28].

In this study it is possible to observe that this condition of severity is expressed as a serious problem within neonatal units' maternal hypertension and infection. This study shows that the group born of hypertensive disease was present in 21.1%, pre-eclampsia in 6.9 %, 4.5%, eclampsia in 7.3% chronic arterial hypertension alone and superimposed on pre-eclampsia and chronic hypertension in 0.6 % and HEELP Syndrome 3.2%.

Commented [MN13]: paragraphs have been deleted or rewritten in order to meet the reviewers' recommendations

Commented [MN14]: References were revised and new ones added with a better relationship with the text and work developed

Studies have pointed to the high risk of adverse outcomes in pregnant women who have overlapping pathologies [3-5]. It was not possible in this study to consider the subtypes diagnosed in the different groups under a more detailed analysis, as the choice was made not to stratify the samples for an individualized analysis of the cases.

However, it was observed that the newborns born to hypertensive mothers had lower birth weight (1054.2 ± 377.3 grams) compared to controls (1321.8 ± 352.5 grams p = 0.00). The same occurred with head circumference (27.8 ±4.2 cm in G1 and 28.8 cm ± 3.2 in G2, p <0.00). In addition, infants born to hypertensive mothers had a higher frequency of intrauterine growth restriction, with 23.7% being classified as small for gestational age (SGA), compared to the control group, at 10.1% (OR 2.4; 95% CI 1.6 – 3.6). These findings are in accordance with studies that found the same association between maternal arterial hypertension and NB with growth restriction [5,12,28].

The weight at discharge, showed an asymmetrical distribution. In this context, the median observed in G1 1970 grams and G2 1980 grams with p = 0.75; however, there was no significant difference between both groups.

When evaluating the babies again, this time at 18 months of age, an attempt was made to identify whether gestational hypertensive disease would be a risk factor for growth disorders in low-weight preterm infants. For this, logistic regression models were constructed, controlled by gestational age and sex, including maternal hypertension. In the classification between birth weight and gestational age, it was observed that HDP was not a risk factor for inadequate weight increase (OR = 0.54; 95% CI: 0.21 - 2.1) at 18 months of corrected age. Similar data were observed by Kiy et al. [28], in a cohort of premature infants at 18 months.

It was identified in the present study that gestational arterial hypertension increased the chance of small NB for gestational age (SGA) by approximately two and a half times (OR = 2.4; 95% CI: 1.6 – 3.6).

Considering this was a retrospective study, there was a difficulty in finding adequate records of height. The ordering of the main results of this study alerts us to the higher frequency of growth disorder in low-birth-weight preterm children born to hypertensive mothers [8,10,11].

There were no differences regarding the need for resuscitation and the diagnosis of respiratory distress syndrome, or the need for exogenous surfactant. Although a significantly increased risk for RDS was not found, some authors suggest that this type of association is possible [10,11].

Maternal hypertension and prematurity can lead to immaturity in the fetal gastrointestinal tract, poor vascular supply, and alteration of the intestinal microbiota, which in turn can be associated with a cascade of events, culminating in the development of necrotizing enterocolitis (NEC) [29,30]. In the present study, a significant association was observed between hypertensive maternal disease and neonatal NEC OR of 3.8 (95% CI 1.7 - 8.6),

There were no differences between the groups regarding the time of use of mechanical ventilation, nasal CPAP and oxygen at 28 days or 36 weeks of gestational age, demonstrating that respiratory changes are not linked to maternal pressure changes, but to the complications and limitations of prematurity and low birth weight.

The literature is conflicting about the effects of pre-eclampsia and the development of retinopathy of prematurity (ROP), one of the main causes of childhood blindness worldwide. No differences in the diagnosis of ROP between groups were observed in this study [32]. Retinopathy of prematurity is initially manifested with delayed physiological vascular development of the retina, followed by aberrant vasoproliferation, and is highly correlated with extreme prematurity and low postnatal growth. [32,33].

In the studied sample, there were no differences between groups regarding the presence of cerebral hemorrhage, however we observed that leukomalacia was more frequent in G1 OR 3.1 CI 95% 1.1 - 8.8. These changes can be identified by brain ultrasound and are associated with an increased risk of neurodevelopmental disorders, which may inform the need for greater pediatric surveillance of babies exposed to maternal hypertensive disease. Ensuring early stimulation can help improve the outcome of neurological development; therefore, the neurological prognosis for these premature babies whose mothers have high blood pressure is still very controversial [10,12].

Although the diagnosis of early neonatal sepsis is more frequent in G2, no statistical difference was observed between groups OR 0.8 of CI 95% 0.6 - 1.1; the same was found in the diagnosis of late sepsis, which was more frequent in G1, but without statistical difference OR 1.3 of CI 95% 1.0 - 1.8.

Higher neonatal mortality was observed in the group of hypertensive mothers, confirming the results of previous studies [8,10,11,34,35]. When the results were adjusted for birth weight, the presence of hypertension was not shown to be an isolated risk factor for death (OR 1.3 of 95% CI 0.8 - 1.9). This result follows what has been described in the literature.

Commented [MN15]: In an attempt to meet the recommendations, the discussion was completely rewritten

There are important limitations of this study that must be considered when interpreting the reported findings. As the project was of a retrospective nature, suggesting that other confounding factors may be operating and have not been identified. However, it was still possible to detect significant and clinically relevant differences between preterm infants born to hypertensive mothers compared to normotensive mothers.

The results of this study show that gestational hypertensive disorder has short-term repercussions in premature newborns, who are born more frequently by cesarean section and are at greater risk of being born small for gestational age and of presenting necrotizing enterocolitis. Studies with a larger sample size of a multicentric and prospective nature with late follow-up are necessary to consolidate these findings.

In these NB studies, it is possible to see that the population of newborns with Hypertensive Disorders of Pregnancy treated at the HMIB had more mortality and morbidity. We recommend careful monitoring of hypertensive pregnant women with a multidisciplinary and careful approach, guiding the follow-up of these women and their children to improve the clinical results of these newborns.

Acknowledgements

We gratefully appreciate the support of medical doctors in the Neonatal Intensive Care Unit of the Brasília Mother and Child Hospital.

Conflict of interest

The authors declare that they have no conflict of interests.

Author contributions

All authors were involved in the concept and design of the study. PRM and MRCGN conducted independent literature searches and quality assessment; MDRM performed data extraction and wrote the first draft of the manuscript. KN and PRM supervised the article selection, quality assessment and data presentation.

All authors have read and approved the final manuscript and agree to be accountable for all aspects of the work. No form of payment was given to anyone to produce the manuscript.

Funding information

Not applicable.

References

1. Shah S, Gupta A. Hypertensive disorders of pregnancy. *Cardiol Clin* 2019; **37**: 345–354.
2. Umesawa M, Kobashi G. Epidemiology of hypertensive disorders in pregnancy: prevalence, risk factors, predictors and prognosis. *Hypertens Res* 2017; **40**: 213–220.
3. Brown MA, Magee LA, Kenny LC, Karumanchi SA, McCarthy FP, Saito S, *et al.* The hypertensive disorders of pregnancy: ISSHP classification, diagnosis & management recommendations for international practice. *Pregnancy Hypertens* 2018; **13**: 291–310.
4. Yu H, He Y, Mao Z, Dong W, Fu X, Lei X. Hypertensive disorders during pregnancy and elevated blood pressure in the offspring: a systematical review and meta-analysis protocol. *Medicine* 2019; **98**: e15677.
5. Nzelu D, Dumitrascu-Biris D, Kay P, Nicolaides KH, Kametas NA. Severe hypertension, preeclampsia and small for gestational age in women with chronic hypertension diagnosed before and during pregnancy. *Pregnancy Hypertens* 2018; **14**: 200–204.
6. Rodrigues Â, Barata C, Marques I, Almeida MC. Diagnosis of white coat hypertension and pregnancy outcomes. *Pregnancy Hypertens* 2018; **14**: 121–124.
7. Wang Y, Hao M, Sampson S, Xia J. Elective delivery versus expectant management for pre-eclampsia: a meta-analysis of RCTs. *Arch Gynecol Obstet* 2017; **295**: 607–622.
8. Omani-Samani R, Ranjbaran M, Amini P, Esmailzadeh A, Sepidarkish M, Almasi-Hashiani A. Adverse maternal and neonatal outcomes in women with preeclampsia in Iran. *J Matern Fetal Neonatal Med* 2019; **32**: 212–216.
9. Nisa MU, Anjum S. Elective induction versus expectant management of mild pre-eclampsia at term. *J Coll Physicians Surg Pak* 2018; **28**: 677–680.
10. Marins LR, Anizelli LB, Romanowski MD, Sarquis AL. How does preeclampsia affect neonates? Highlights in the disease's immunity. *J Matern Fetal Neonatal Med* 2019; **32**: 1205–1212.
11. O'Gorman N, Nicolaides KH, Poon LC. The use of ultrasound and other markers for early detection of preeclampsia. Womens Health (Lond). 2016;**12**(2):199-207.

12. Maher GM, O'Keeffe GW, Kearney PM, Kenny LC, Dinan TG, Mattsson M, *et al.* Association of hypertensive disorders of pregnancy with risk of neurodevelopmental disorders in offspring: a systematic review and meta-analysis. *JAMA Psychiatry* 2018; **75**: 809–819

13. Nathan HL, Seed PT, Hezelgrave NL, De Greeff A, Lawley E, Conti-Ramsden F, *et al.* Maternal and perinatal adverse outcomes in women with pre-eclampsia cared for at facility-level in South Africa: a prospective cohort study. *J Glob Health* 2018; **8**: 020401.

14. von Elm E, Altman DG, Egger M, Pocock SJ, Gotzsche PC, Vandenbroucke JP, *et al.* The strengthening the reporting of observational studies in epidemiology (STROBE) statement: guidelines for reporting observational studies. *J Clin Epidemiol* 2008; **61**: 344–349.

15. Ballard JL, Khoury JC, Wedig K, Wang L, Eilers-Walsman BL, Lipp R. New Ballard Score, expanded to include extremely premature infants. *J Pediatr*. 1991;**119**: 417-423.

16. Lubchenco L, Hansman C, Dressler M, Boyd E. Intrauterine growth as estimated from liveborn birth-weight data at 24 to 42 weeks of gestation. *Pediatrics* 1963; **32**: 793–800.

17. Margotto PR. Curvas de crescimento intra-uterino: estudo de 4413 recém-nascidos únicos de gestações normais. *J Pediatr* 1995; **71**: 11–21.

18. Papile LA, Burstein J, Burstein R, Koffler H. Incidence and evolution of subependymal and intraventricular hemorrhage: A study of infants with birth weights less than 1,500 gm. *J Pediatr* 1978; **92**: 529–534.

19. Villar J, Carroli G, Wojdyla D, Abalos E, Giordano D, Ba'aqeel H, *et al.* Preeclampsia, gestational hypertension and intrauterine growth restriction, related or independent conditions? *Am J Obstet Gynecol* 2006; **194**: 921–931.

20. Webster LM, Conti-Ramsden F, Seed PT, Webb AJ, Nelson-Piercy C, Chappell LC. Impact of antihypertensive treatment on maternal and perinatal outcomes in pregnancy complicated by chronic hypertension: a systematic review and meta-analysis. *J Am Heart Assoc* 2017; **6**: e005526.

21. Shen M, Smith GN, Rodger M, White RR, Walker MC, Wen SW. Comparison of risk factors and outcomes of gestational hypertension and pre-eclampsia. *PLoS One* 2017; **12**: e0175914.

22. Deshmukh M, Patole S. Antenatal corticosteroids for neonates born before 25 weeks-a systematic review and meta-analysis. *PLoS One* 2017; **12**: e0176090.

23. Duley L, Gülmezoglu AM, Henderson-Smart DJ, Duley L. Magnesium sulphate and other anticonvulsants for women with pre-eclampsia. *Cochrane Database Syst Rev* 2010; **2010**: CD000025.

24. Jameson RA, Bernstein HB. Magnesium sulfate and novel therapies to promote neuroprotection. *Clin Perinatol* 2019; **46**: 187–201.

25. American College of Obstetricians Gynecologists, Task Force on Hypertension in Pregnancy. Hypertension in pregnancy. Report of the American college of obstetricians and gynecologists' task force on hypertension in pregnancy. *Obstet Gynecol* 2013; **122**: 1122–1131.

26. Vogel JP, Chawanpaiboon S, Moller A-B, Watananirun K, Bonet M, Lumbiganon P. The global epidemiology of preterm birth. *Best Pract Res Clin Obstet Gynaecol* 2018; **52**: 3–12.

27. Razak A, Florendo-Chin A, Banfield L, Abdul Wahab MG, McDonald S, Shah PS, *et al.* Pregnancy-induced hypertension and neonatal outcomes: a systematic review and meta-analysis. *J Perinatol* 2018; **38**: 46–53.

28. Gagliardi L, Rusconi F, Bellù R, Zanini R; Italian Neonatal Network. Association of maternal hypertension and chorioamnionitis with preterm outcomes. *Pediatrics*. 2014; **134**:e154-e161.

29. Kiy AM, Rugolo LMSS, Luca AKCD, Corrente JE. Growth of preterm low birth weight infants until 24 months corrected age: effect of maternal hypertension. *J Pediatr* 2015; **91**: 256–262.

29. Yang C-C, Tang P-L, Liu P-Y, Huang W-C, Chen Y-Y, Wang H-P, *et al.* Maternal pregnancy-induced hypertension increases subsequent neonatal necrotizing enterocolitis risk: a nationwide population-based retrospective cohort study in Taiwan. *Medicine* 2018; **97**: e11739.

30. Neu J. Necrotizing enterocolitis. *World Rev Nutr Diet* 2014; **110**: 253–263.

31. Vieira AA, David BBL, Lino RRG, Duarte LDB, Bueno AC. Avaliação dos fatores perinatais que interferem na incidência de enterocolite necrosante em recém-nascidos de muito baixo peso. *Rev Bras Ginecol Obstet* 2013; **35**: 363–367.

32. Zhu T, Zhang L, Zhao F, Qu Y, Mu D. Association of maternal hypertensive disorders with retinopathy of prematurity: a systematic review and meta-analysis. *PLoS One* 2017; **12**: e0175374.

33. Bashinsky AL. Retinopathy of prematurity. *N C Med J* 2017; **78**: 124–128.

34. Ndwiga C, Odwe G, Pooja S, Ogutu O, Osoti A, E Warren C. Clinical presentation and outcomes of pre-eclampsia and eclampsia at a national hospital, Kenya: A retrospective cohort study. *PLoS One*. 2020;**15**:e0233323.

35. Dassah ET, Kusi-Mensah E, Morhe ESK, Odoi AT. Maternal and perinatal outcomes among women with hypertensive disorders in pregnancy in Kumasi, Ghana. *PLoS One*. 2019;**14**: e0223478.

Figure 1. Schematic diagram showing the number of participants medical birth registry, 2014–2019.

Commented [MN16]: the graph has been adjusted and is more objective

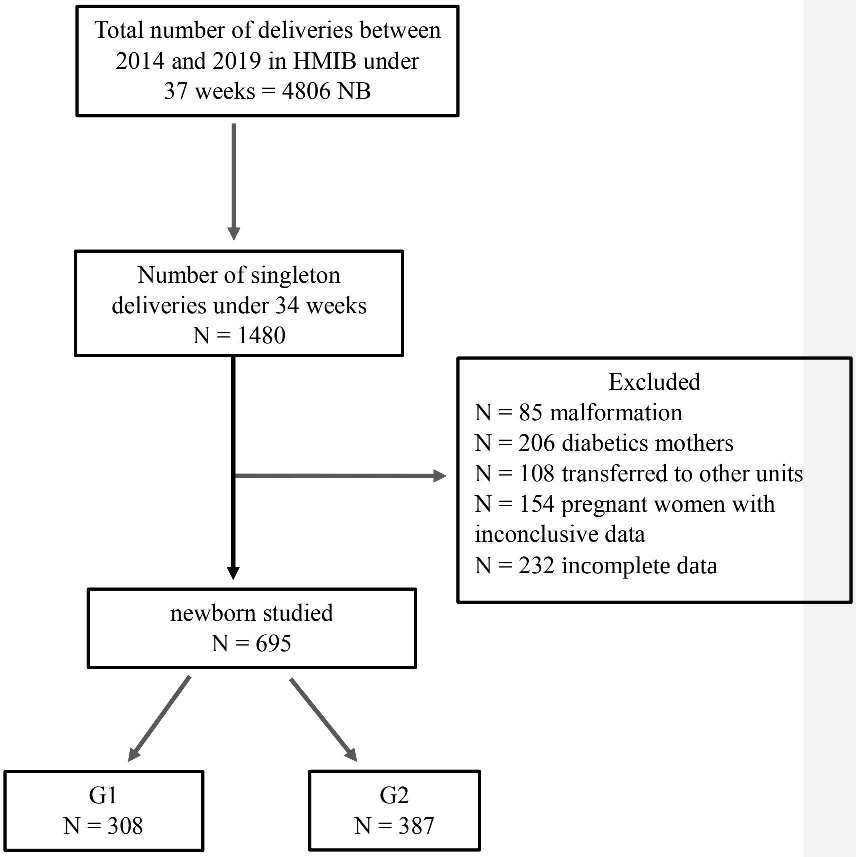

Table 1. Characteristics of the studied pregnant women divided considering the presence or not of Hypertensive disorders of pregnancy.

Commented [MN17]: The tables have been rewritten to meet the recommendations

	G1<br>n: 308 NB	G2<br>n: 387 NB	p	OR<br>(95% CI)
Maternal Age (years)	28.2 ± 6.8	26.1 ± 6.9	0.00	-
Number of prenatal consultations	5.3 ± 2.4	5. ± 2.4	0.63	-
Gestational Age	28.9± 2.9	29.8 ± 2.2	0.00	-
Cesarean delivery%	68.5	34.4	0.00	0.2 (0.2 to 0.3)
Urinary infection %	12.0	23.0	0.00	0.4 (0.3 to 0.7)
Chorioamnionitis%	13.0	25.3	0.00	0.4 (0.3 to 0.6)
Prenatal corticosteroids%	77.3	73.1	0.21	1.2 (0.8 to 1.8)
Magnesium Sulfate%	59.7	36.4	0.00	2.6 (1.9 to 3.5)

Data: mean ± SD, p-Value < 0,05 via Student't – test; Data: n (percent), p – Value < 0,05 via X^2 test

Table 2. Neonates' morbidity considering the presence or not of Hypertensive disorders of pregnancy.

	G1<br>n: 308 NB	G2<br>n: 387 NB	p	OR<br>(95% CI)
Birth weight (grams)	1054.2± 377.3	1321.8 ± 352.5	0.00	-
Head circumference (cm)	27.8 ± 4.2	28.8 ± 3.2	0.00	-
Mechanical ventilation (days)	7.3 ± 15.6	5.2 ± 13.0	0.05	-
Apgar 1 min	5.9 ± 2.1	6.3 ± 2.1	0.02	-
Apgar 5 min	7.8 ± 1.6	7.8 ± 1.5	0.97	-
Hospitalization time in days	37.8 ± 32.3	36.7 ± 39.5	0.76	-
Weight at hospital discharge	1919.5 ± 226.9	1.811.9 ± 346.1	0.00	-
NB <850 g %	23.7	11.4	0.00	2.4 (1.4 to 3.5)
SGA rating%	23.7	10.1	0.00	2.4 (1.6 to 3.6)
Male %	50.3	48.8	0.70	1.0 (0.8 to 1.4)
Neonatal resuscitation %	66.6	61.5	0.17	1.2 (0.9 to 1.7)
Use of surfactant%	47.7	41.6	0.10	1.3 (0.9 to 1.7)
Early sepsis%	34,1	39.3	0.17	0.8 (0.6 to 1.1)
Later sepsis%	37.7	31.0	0.07	1.3 (1.0 to 1.8)
Use of O2 at 36 weeks%	23.0	24.4	0.81	0.9 (0.6 to 1.5)
Necrotizing Enterocolitis%	8.4	4.4	0.03	2.0 (1.1 to 3.7)
Retinopathy of prematurity%	13.6	15.0	0,63	0.8 (0.5 to 1.4)
Leukomalacia (%)	6.3	2.1	0.03	3.1 (1.1 to 8.8)

HIV: Intraventricular Hemorrhage, Data: n (percent), p – Value< 0,05 via X^2 test
Data: mean ± SD, p-Value < 0,05 via Student't – test; NB – New Born; SGA - Small for gestational age.

Commented [MN18]:

Table 3. Survival at discharge and at 18 months in neonates considering the presence or not of Hypertensive disorders of pregnancy.

	G1 n: 308 RN	G2 n: 387 RN	p	OR (IC 95%)	
Survival at discharge (%)	69.8	77.3	0.03	0.7	(0.5 to 0.9)
Exclusive breastfeeding at discharge (%)	73.4	77.7	0.25	0.8	(0.5 to 1.1)
Survival at 18 months (%)	95.3	92.1	0.15	1.7	(0.9 to 3.7)
Breastfeeding at 18 months (%)	66.7	70.9	0.28	0.8	(0.6 to 1.1)
Weight at 18 months (kg)	8.868 ± 0.720	8.830 ± 0.670	0.72		

Data: mean ± SD, p-Value < 0,05 via Student't – test / Data: n (percent), p – Value< 0,05 via X^2 test
