# Supplementary material for: Hypertension induced by pregnancy and neonatal outcome: Results from a retrospective cohort study in preterm under 34 weeks
Source: PLoS One. 2021 Aug 18;16(8):e0255783. doi: 10.1371/journal.pone.0255783 (PMC8372928; doi:10.1371/journal.pone.0255783)
Supplement: Attachment — Submitted filename: Response to Reviewers.docx [file pone.0255783.s003.docx]

Dear Editor:

I am pleased to resubmit for publication the revised version of: “Hypertension induced by pregnancy and neonatal outcome: Results from a retrospective cohort study in preterm under 34 weeks.” , PLOS ONE-D-20-32227R1, under the authorship of , Marta David Rocha de Moura, Paulo Roberto Margotto, Karina Nascimento and Maria Rita CG Novaes for your appreciation.

First, we would like to thank the reviewers of this study who provided valuable suggestions and very pertinent comments, thus helping to improve the quality of our article. After a detailed analysis of the comments and questions, as well as the errors pointed out and suggestions contained in the opinions sent to us, the article underwent some changes, which are indicated in the archive Manuscript with Track Changes.

Thank you for your consideration!

Sincerely,

Marta David Rocha de Moura, M.D

Assistant doctor, neonatal intensive care unit, Brasília Mother and Child Hospital
